# Advanced machine learning-based screening for primary aldosteronism with plasma steroids, potassium, and renin

**DOI:** 10.1038/s41746-026-02906-w

**Published:** 2026-06-24

**Authors:** Wenyu Zhang, Christina Pamporaki, René Jäkel, Georgiana Constantinescu, Mirko Peitzsch, Manuel Schulze, Jun Yang, Andrea Rita Horvath, Ralph Müller-Pfefferkorn, Tracy Ann Williams, Martin Reincke, Felix Beuschlein, Carmina Teresa Fuss, Stefanie Hahner, Graeme Eisenhofer

**Affiliations:** 1 Center for Scalable Data Analytics and Artificial Intelligence Dresden/Leipzig, Technische Universität, Dresden, Germany; 2 Department of Medicine III, University Hospital Carl Gustav Carus, Technische Universität, Dresden, Germany; 3 Center for Information Services and High Performance Computing, Technische Universität Dresden, Dresden, Germany; 4 Institute of Clinical Chemistry and Laboratory Medicine, University Hospital Carl Gustav Carus, Technische Universität, Dresden, Germany; 5 Centre for Endocrinology and Reproductive Health, Hudson Institute of Medical Research and Department of Medicine, Monash University, Clayton, VIC Australia; 6 New South Wales Health Pathology, Prince of Wales Hospital, Sydney, NSW Australia; 7 Department of Medicine IV, University Hospital, Ludwig Maximilian University Munich, Munich, Germany; 8 Department of Endocrinology, Diabetology and Clinical Nutrition, University Hospital Zurich and University of Zurich and the LOOP Zurich Medical Research Center, Zurich, Switzerland; 9 Department of Internal Medicine I, Division of Endocrinology and Diabetes, University Hospital, University of Würzburg, Würzburg, Germany

**Keywords:** Cardiology, Diseases, Health care, Medical research

## Abstract

Commonly used screening tests for primary aldosteronism (PA) provide suboptimal diagnostic accuracy, particularly with antihypertensive medication use. This study utilized three datasets totaling 1380 patients with and without PA to develop machine learning models for screening based on plasma steroids, potassium, and renin. A feedforward neural network (FNN) model with steroids and potassium improved diagnostic accuracy compared to models without potassium. Inclusion of renin negligibly improved accuracy. The FNN and other renin-independent models showed similar accuracy before and after antihypertensive medication washout, whereas renin-dependent models exhibited poorer accuracy without medication washout. Three further optimized renin-independent models outperformed the aldosterone-to-renin ratio (ARR) for screening according to areas under receiver-operating-characteristic curves of 0.948–0.954 versus 0.839 for the ARR. Those models minimize need for medication washout and, at cut-offs for optimal 90–95% diagnostic sensitivity, reduce false positives by 53–72% to more effectively screen for PA than with the ARR.

## Introduction

Primary aldosteronism (PA), the most frequent cause of secondary hypertension, is characterized by inappropriately high plasma aldosterone concentrations that fail to suppress with volume expansion. Screening traditionally depends on measurements of the plasma aldosterone-to-renin ratio (ARR), a test first introduced by Hiramatsu et al in 1981^1^. Cut-off values for the ARR are optimized to achieve high diagnostic sensitivity, which minimizes the likelihood of missing patients with PA; however, this leads to high false-positive rates of up to 43%^2–5^. Impacts of antihypertensive medications that affect the ARR, particularly through effects on renin, also have to be considered^6–10^. Although interfering antihypertensive drugs reduce diagnostic accuracy of the ARR, they have minimal impact on steroidomics-based diagnostics^6^, which offers a potential alternative to the ARR.

Effective interpretation of multidimensional steroidomics data for diagnostics requires advanced mathematical approaches that can be achieved by machine learning (ML), which recognizes patterns in data rather than simply identifying deviations from predefined reference intervals^11–14^. Lin et al. focused on the application of an online ML model based on the seven clinical characteristics to predict risk of PA^15^. Buffolo et al. developed a scoring system and random forest regressor model to screen for PA^16^. These models highlighted the significance of sex and serum potassium to identify patients with PA but offered limited diagnostic performance and did not include plasma steroid profiles.

Reel and colleagues developed an ML pipeline to distinguish endocrine from primary hypertension using multi-omics data^17^. Among models tested, the multi-omics-based classifier was superior to others for identifying patients with PA among those with other causes of hypertension. However, the study was limited by a small sample size relative to its multi-omics nature. Many models demonstrated superb performance during training but showed a sharp decline during internal testing, likely due to overfitting.

In the Steroid Profiling for Identification and Subtype Classification of Primary Aldosteronism (SPISCA) study, ML models were developed from mass spectrometry-based steroid profiles and adjustments for sex and age to diagnose and classify different subtypes of PA^18,19^. Although these models show promise for subtyping, their accuracy for initial screening remains suboptimal. We therefore determined whether combining renin and potassium measurements with steroid profiles could enhance the performance of ML models for screening before subtype classification. We also examined impacts of antihypertensive medications to assess best models to minimize need for medication washout. To address these objectives, we utilized data from the SPISCA cohort for model training and an independent prospective cohort for external validation. To further optimize model generalizability and robustness, we then integrated both cohorts for training and internal validation and performed external validation using a third independent cohort unknown to ML models. The final objective was to establish whether ML models offer improved diagnostic performance compared to traditional use of the ARR.

## Results

### Workflow, patient cohorts, and characteristics

**Fig. 1:**
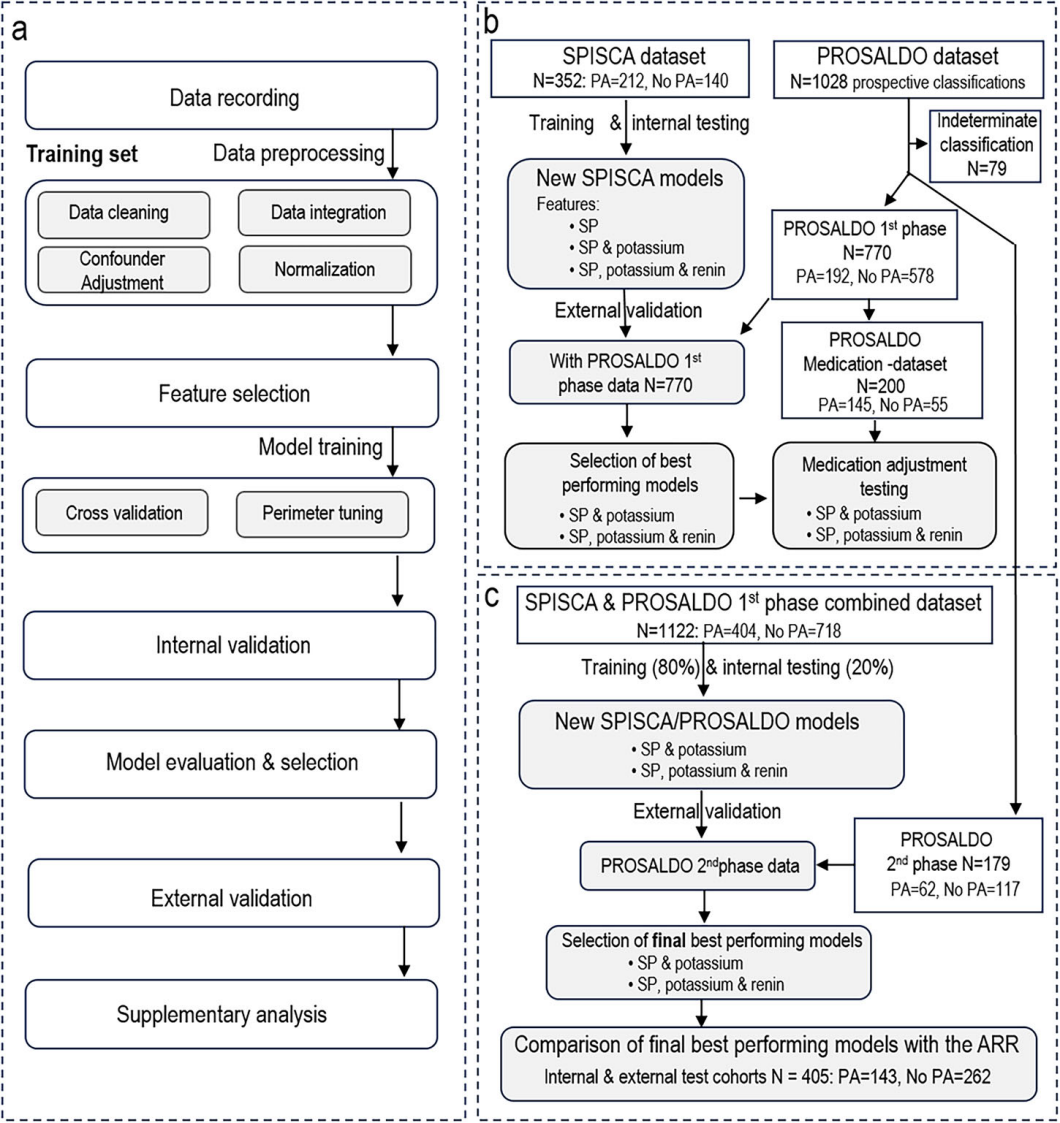
Machine learning workflow and cohort allocation for model development and validation. Panel **a** shows a schematic overview of the end-to-end machine learning pipeline, including data preprocessing, feature selection, model training, internal testing, external validation, and supplementary analysis. Panels **b** and **c** show the cohort-based study design used to develop and validate prediction models using steroid profiles (SP) for screening primary aldosteronism (PA). Panel **b** covers initial model training using the SPISCA cohort, with external validation and examination of impacts of antihypertensive medications using the first phase of the PROSALDO cohort. Panel **c** covers final model development using both SPISCA and first phase PROSALDO cohorts, with external validation using the second phase PROSALDO cohort and final comparisons of diagnostic performance of best models with the aldosterone:renin ratio (ARR).

ML models were developed according to the workflow and patient cohorts illustrated in Fig. 1. The pipeline included data preprocessing, feature selection, and model training (Fig. 1a). Developed ML models were internally tested and then externally validated in independent datasets.

A total of 1380 patients were included from three patient cohorts: (i) 352 patients from the SPISCA dataset, including 212 and 140 respective patients with and without PA in whom all results for steroids, potassium and renin were available; (ii) 849 patients from the first phase of the PROspective study on the diagnostic value of Steroid profiling in primary ALDOsteronism (PROSALDO), including 192 and 578 with and without PA; and (iii) 179 patients from the second phase of the PROSALDO study, including 62 and 117 patients with and without PA. Classifications were not possible in 79 patients, who were therefore excluded from model development and testing.

Model development was initially restricted to the smaller SPISCA cohort in order to subsequently employ the PROSALDO cohort to assess differences in model performance and impacts of antihypertensive medications (Fig. 1b). The third cohort was employed to externally validate subsequently optimized ML models developed from combined SPISCA and PROSALDO cohorts, which were also used to compare diagnostic performance of models for screening against traditional use of the ARR (Fig. 1c).

As detailed in Table 1, patients in SPISCA and first phase PROSALDO cohorts showed some differences in clinical and biochemical characteristics to support generalizability of models developed using the SPISCA dataset. As expected, there were lower renin and potassium concentrations and higher plasma concentrations of mineralocorticoids and some glucocorticoids in patients with than without PA in both SPISCA and PROSALDO cohorts (Supplementary Table 1).

**Table 1:** Clinical and biochemical characteristics of patients in the SPISCA and PROSALDO cohorts used for machine learning modeling

PA				No PA		
	SPISCA	PROSALDO	*P*-value	SPISCA	PROSALDO	*P*-value
Patient demographics						
*N*	212	192		140	578	
Sex (F/M)	90/122	80/112	0.8714	72/68	320/258	0.3966
Age (years)	49.8 (48.2–51.4)	48.8 (47.2–50.5)	0.3604	44.6 (42–47.3)	47.1 (46–48.2)	0.2261
Routine measurements						
Potassium (mmol/L)	3.31 (3.22–3.4)	3.62 (3.55–3.69)	<0.0001	4.03 (3.96–4.1)	4.19 (4.15–4.22)	0.0002
Renin (mU/L)	3.76 (3.37–4.2)	4.62 (4.06–5.25)	0.0985	12.12 (10.02–14.66)	11.01 (10.08–12.03)	0.3135
LC–MS/MS Steroids (ng/mL)						
Aldosterone	0.183 (0.166–0.203)	0.200 (0.184–0.218)	0.0394	0.063 (0.056–0.072)	0.071 (0.067–0.075)	0.0633
18-Oxo-cortisol	0.042 (0.034–0.051)	0.053 (0.045–0.064)	0.0112	0.014 (0.012–0.015)	0.015 (0.014–0.016)	0.1854
18-Hydroxycortisol	1.101 (0.974–1.244)	1.124 (1.009–1.253)	0.8394	0.617 (0.561–0.678)	0.623 (0.599–0.648)	0.771
11-Deoxycorticosterone	0.086 (0.075–0.099)	0.057 (0.051–0.064)	<0.0001	0.039 (0.034–0.044)	0.030 (0.028–0.031)	<0.0001
Corticosterone	3.01 (2.63–3.45)	2.25 (2.03–2.5)	0.0023	2.33 (2.02–2.69)	1.87 (1.76–1.98)	0.0042
Cortisol	121 (113–130)	104 (99–110)	0.0001	132 (122–142)	101 (98–104)	<0.0001
Cortisone	17.3 (16.3–18.3)	17.7 (17.0–18.4)	0.8958	19.8 (18.9–20.8)	17.8 (17.5–18.2)	<0.0001
11-Deoxycortisol	0.419 (0.373–0.472)	0.337 (0.305–0.371)	0.0111	0.228 (0.2–0.26)	0.224 (0.212–0.226)	0.7907
21-Deoxycortisol	0.022 (0.018–0.027)	0.024 (0.022–0.027)	0.4876	0.012 (0.009–0.016)	0.019 (0.018–0.020)	0.0034
17-Hydroxyprogesterone	0.859 (0.772–0.956)	0.590 (0.533–0.653)	<0.0001	0.542 (0.468–0.628)	0.431 (0.402–0.462)	0.0034
Androstenedione	1.214 (1.124–1.31)	0.666 (0.622–0.714)	<0.0001	1.075 (0.976–1.184)	0.607 (0.582–0.632)	<0.0001
DHEA	1.92 (1.71–2.15)	2.46 (2.23–2.71)	0.0024	2.16 (1.868–2.5)	2.43 (2.29–2.58)	0.2887
DHEAS	968 (882–1062)	925 (833–1026)	0.6313	1168 (1021–1336)	972 (910–1039)	0.0047

Continuous parameters are shown as geometric means with confidence intervals.

### Initial model development from the SPISCA cohort

To assess whether diagnostic performance of models using steroids alone could be improved with inclusion of potassium and renin, we first developed models based on steroids alone using the SPISCA cohort and modified procedures to those used previously (Fig. 1b). Those models were then compared with a support vector machine (SVM) model previously established from the SPISCA cohort^18^. Six best models based on steroids were selected for external validation using data from the first phase PROSALDO cohort. Models incorporated from three to six selected steroids compared to seven in the previously established SVM model (Supplementary Table 2). Based on metrics of balanced accuracy, F1 scores, Matthews correlation coefficient, and areas under receiver-operating characteristic curves (AUC), there were two best models developed using feedforward neural network (FNN) and SVM algorithms, which were followed by models developed using multilayer perceptron (MLP), random forest (RF), logistic regression (LR) and Gaussian Naïve Bayes (GNB) algorithms (Table 2). According to AUCs, all displayed similar performance to the previously developed SVM model.

**Table 2:** Evaluation metrics for classification performance of machine learning models trained on the SPISCA cohort after external validation on the PROSALDO cohort

Model	Balanced Accuracy	F1 score	MCC	AUC	*P* value*
Previous model					
SVM	0.821 (0.791–0.894)	0.705 (0.661–0.792)	0.602 (0.557–0.724)	0.917 (0.893–0.937)	-
Steroids only					
SVM	0.833 (0.808–0.877)	0.727 (0.669–0.814)	0.630 (0.550–0.756)	0.920 (0.892–0.947)	0.6892
FNN	0.841 (0.813–0.884)	0.742 (0.65–0.806)	0.651 (0.563–0.740)	0.923 (0.895–0.948)	0.3719
MLP	0.837 (0.809–0.884)	0.709 (0.675–0.8)	0.608 (0.56–0.741)	0.917 (0.885–0.945)	0.9778
LR	0.833 (0.808–0.876)	0.719 (0.663–0.790)	0.618 (0.547–0.717)	0.914 (0.885–0.943)	0.6764
RF	0.824 (0.797–0.872)	0.736 (0.658–0.803)	0.649 (0.535–0.742)	0.913 (0.884–0.939)	0.6349
GNB	0.820 (0.784–0.863)	0.712 (0.643–0.78)	0.609 (0.512–0.706)	0.902 (0.868–0.931)	0.1033
Steroids + Potassium					
SVM	0.864 (0.833–0.899)	0.778 (0.730–0.843)	0.700 (0.638–0.789)	0.939 (0.916–0.960)	0.0138
FNN	0.866 (0.840–0.907)	0.795 (0.746–0.861)	0.725 (0.657–0.815)	0.940 (0.918–0.963)	0.0103
MLP	0.865 (0.837–0.905)	0.782 (0.731–0.843)	0.707 (0.639–0.791)	0.938 (0.916–0.961)	0.0235
LR	0.849 (0.823–0.889)	0.761 (0.689–0.821)	0.678 (0.583–0.763)	0.935 (0.910–0.956)	0.0691
RF	0.856 (0.825–0.899)	0.771 (0.725–0.837)	0.692 (0.631–0.783)	0.929 (0.906–0.953)	0.1888
GNB	0.839 (0.812–0.881)	0.739 (0.679–0.816)	0.647 (0.566–0.757)	0.924 (0.898–0.951)	0.4416
Steroids + Potassium + Renin					
SVM	0.885 (0.859–0.913)	0.770 (0.732–0.828)	0.696 (0.642–0.773)	0.951 (0.932–0.968)	0.0024
FNN	0.876 (0.853–0.911)	0.770 (0.726–0.848)	0.692 (0.637–0.796)	0.951 (0.931–0.968)	0.0033
MLP	0.872 (0.847–0.911)	0.784 (0.725–0.847)	0.708 (0.635–0.795)	0.947 (0.924–0.967)	0.0049
LR	0.877 (0.857–0.911)	0.747 (0.723–0.821)	0.670 (0.634–0.759)	0.950 (0.931–0.966)	0.0023
RF	0.861 (0.828–0.893)	0.748 (0.705–0.800)	0.661 (0.600–0.733)	0.935 (0.914–0.955)	0.1132
GNB	0.883 (0.857–0.915)	0.784 (0.741–0.845)	0.710 (0.652–0.792)	0.952 (0.933–0.969)	0.0011

*All selected models were evaluated for significant improvement over the previously established SVM model using a paired AUC test.

*FNN* feedforward neural networks, *GNB* Gaussian naïve Bayes, *MLP* multi–layer perceptron, *LR* logistic regression, *SVM* support vector machine, *RF* random forest, *MCC* Matthews correlation coefficient. The confidence interval for each evaluation scoring was calculated via bootstrapping.

We then developed models using serum potassium and plasma steroids, from which six best new models were selected for external validation. Besides potassium, all models selected aldosterone, 18-oxocortisol, and 11-deoxycorticosterone as critical features (Supplementary Table 2). From feature weight analysis, using assessments of permutation importance (PI), aldosterone and 18-oxocortisol were the top two features for all models except the LR model (Supplemental Fig. 1). With external validation, inclusion of potassium increased (*P* < 0.05) the AUC from 0.917 for the previously established SVM model to 0.940, 0.938 and 0.939 for respective top-performing FNN, MLP and SVM models (Table 2).

After further development of models trained also with renin, six were again selected for external validation. The FNN and GNB models selected only two steroids, aldosterone and 18-oxocortisol, as features (Supplementary Table 2). The other four renin-dependent models—SVM, MLP, LR, and RF—selected 11-deoxycorticosterone or 11-deoxycortisol as additional essential features, albeit with generally lower PI values than for other selected features (Supplementary Fig. 1). Apart from the RF model, all renin-dependent models exhibited similar performance according to AUCs of 0.947–0.952 that were higher (*P* < 0.005) than the AUC for the previously established SVM model (Table 2).

### Impacts of antihypertensive medications

**Fig. 2:**
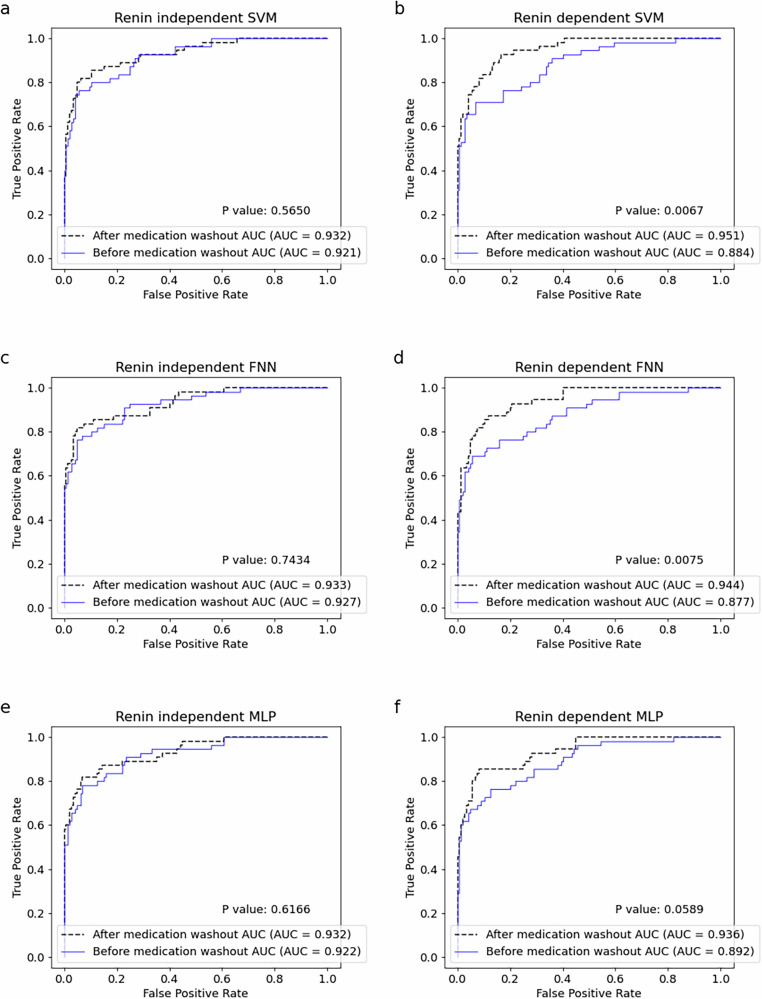
Effect of antihypertensive medication washout on the diagnostic performance of machine learning models. Panels show receiver operating characteristic (ROC) curves for support vector machine (SVM) (**a**, **b**), feedforward neural network (FNN) (**c**, **d**), and multilayer perceptron (MLP) (**e**, **f**) renin-independent (**a**, **c**, **e**) and renin-dependent (**b**, **d**, **f**) models before and after antihypertensive medication washout. Blue solid lines represent performance before medication washout, and black dashed lines represent performance after washout. Areas under ROC curves (AUCs) are indicated in each panel. *P* values refer to pairwise comparisons of AUCs before and after medication washout.

We then evaluated whether antihypertensive medications known to confound interpretation of the ARR for screening also impact diagnostic performance of renin-independent and renin-dependent ML models (Fig. 1b). For this, we used data from 200 patients of the PROSALDO cohort, including 55 and 145 respective patients with and without PA (Supplementary Table 3), in whom data were available before and after antihypertensive medication washout. Renin-independent SVM, FNN, and MLP models, which included steroids and potassium as features, showed similar performance with and without medication washout (Fig. 2a, c, e). In contrast, the renin-dependent SVM and FNN models displayed reduced (*P* < 0.01) diagnostic performance without compared to with medication washout (Fig. 2b, d). The MLP model was the only renin-dependent model in which the drop in diagnostic performance did not reach significance (*P* = 0.0589) (Fig. 2f).

### Final model development using combined SPISCA & PROSALDO cohorts

For final development of renin-independent and renin-dependent ML models we employed combined SPISCA and PROSALDO cohorts (Fig. 1c). Model training and internal testing involved a total of 1,122 patients, among whom 896 (80%) were used for training, and 226 (20%) were reserved for internal testing. Models were then externally validated using the second phase PROSALDO dataset of 62 and 117 patients with and without PA, respectively.

Six renin-independent models were developed using plasma steroid concentrations and serum potassium, while inclusion of renin provided for the renin-dependent models. In contrast, all renin-dependent models selected two to three steroids in addition to potassium and renin. All models selected aldosterone as the top steroid feature, and most others, except the LR model selected 18-oxocortisol as the next top steroid (Table 3). Potassium showed high PI values across all models, including renin-dependent models for which PI values were higher than for renin (Supplementary Fig. 2).

**Table 3:** Normalizations and feature selection methods for the renin-independent and renin-dependent machine learning models trained and tested on combined cohorts

Model	Normalization	Feature selection	Selected features	Steroid number
Renin-independent model				
SVM	Min-max	RFE	ALD K 18OXOF AE DOC E	5
FNN	Min-max	RFE	ALD K 18OXOF DOC AE E	5
MLP	Min-max	RFE	ALD K 18OXOF AE E DOC	5
LR	Min-max	RFE	ALD K AE DOC E 18OXOF F DHEA	7
RF	Quantile	SKB	ALD K 18OXOF DOC	3
GNB	Min-max	SKB	K ALD 18OXOF DOC	3
Renin-dependent model				
SVM	Min-max	RFE	ALD K 18OXOF Renin AE	3
FNN	Robust	SKB	ALD K Renin 18OXOF DOC	3
MLP	Standard	SFM	ALD K 18OXOF Renin	2
LR	Robust	RFE	ALD K Renin AE E	3
RF	Min-max	RFE	ALD 18OXOF K Renin AE	3
GNB	Min-max	RFE	ALD K 18OXOF Renin AE	3

*FNN* feedforward neural networks, *GNB* Gaussian naïve Bayes, *MLP* multi-layer perceptron, *LR* logistic regression, *SVM* support vector machine, *RF* random forest, *Standard* standardization, *Min-max* min-max scaling, *Robust* robust scaling, *SKB* SelectKBest, *RFE* recursive feature elimination, *SFM* SelectFromModel, *ALD* aldosterone, *18OXOF* 18-oxocortisol, *18OHF* 18-hydroxycortisol, *21DF* 21-deoxycortisol, *CORT* corticosterone, *E* cortisone, *DHEA* dehydroepiandrosterone, *DHEAS* DHEA sulfate, *DOC* 11-deoxycorticosterone, *S* 11-deoxycortisol, *17-OHP* 17-hydroxyprogesterone, *F* cortisol, *AE* androstenedione, *K* Potassium.

**Fig. 3:**
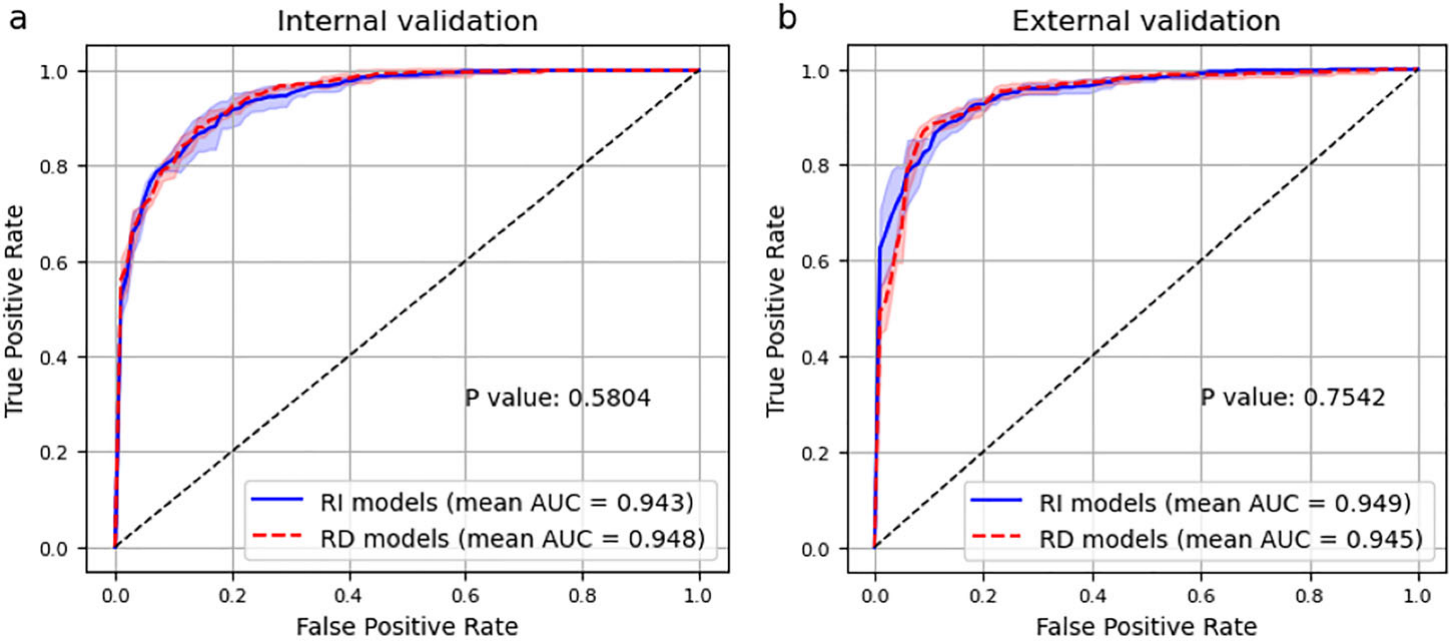
Comparison of renin-independent and renin-dependent machine learning models. Panels **a** and **b** show receiver operating characteristic (ROC) curve comparisons of renin-independent (RI) and renin-dependent (RD) machine learning models during internal (**a**) external (**b**) validation using combined SPISCA and PROSALDO cohorts. Solid blue lines indicate renin-independent models, and dashed red lines indicate renin-dependent models. Shaded areas indicate variability across models within each group. Areas under ROC curves (AUCs) are shown, and *P* values refer to statistical comparisons between renin-independent and renin-dependent model performance.

Using the metrics of balanced accuracy, F1 scores, Matthews correlation coefficient, and AUCs, the FNN, MLP and SVM models were again established to represent the top-performing renin-independent models according to both internal (Supplementary Table 4) and external (Table 4) validation datasets. Those models were followed by the RF and LR models, and again, the GNB as the poorest performer of all six models. Renin-dependent models showed variable differences in performance for internal and external validation datasets, though overall the SVM and MLP models were the top-performers followed by RF, GNB, FNN, and LR models. Performance of renin-independent and renin-dependent models was similar across validations, with average AUCs of 0.949 and 0.945, respectively, for external validation (Fig. 3a, b).

**Table 4:** Evaluation metrics for classification performance of machine learning models trained on the SPISCA and PROSALDO combined cohort after external validation

Model	Balanced accuracy	F1 score	MCC	AUC
Renin-independent models				
SVM	0.900 (0.875–0.929)	0.857 (0.825–0.901)	0.778 (0.728–0.849)	0.952 (0.928–0.971)
FNN	0.902 (0.874–0.928)	0.876 (0.836–0.91)	0.813 (0.724–0.866)	0.957 (0.938–0.974)
MLP	0.894 (0.870–0.925)	0.867 (0.827–0.904)	0.801 (0.733–0.857)	0.955 (0.935–0.971)
LR	0.884 (0.857–0.913)	0.844 (0.805–0.88)	0.758 (0.695–0.816)	0.944 (0.922–0.962)
RF	0.878 (0.85–0.91)	0.826 (0.790–0.867)	0.729 (0.671–0.795)	0.944 (0.921–0.963)
GNB	0.872 (0.831–0.890)	0.828 (0.785–0.867)	0.733 (0.671–0.794)	0.943 (0.921–0.961)
Renin-dependent models				
SVM	0.906 (0.883–0.935)	0.878 (0.839–0.913)	0.814 (0.753–0.867)	0.945 (0.921–0.964)
FNN	0.901 (0.870–0.928)	0.866 (0.819–0.902)	0.793 (0.722–0.850)	0.945 (0.923–0.963)
MLP	0.905 (0.875–0.932)	0.873 (0.836–0.910)	0.804 (0.747–0.861)	0.947 (0.924–0.966)
LR	0.888 (0.861–0.917)	0.85 (0.807–0.888)	0.769 (0.702–0.827)	0.945 (0.923–0.962)
RF	0.891 (0.867–0.916)	0.844 (0.814–0.888)	0.758 (0.711–0.831)	0.947 (0.926–0.964)
GNB	0.897 (0.868–0.923)	0.859 (0.815–0.894)	0.782 (0.716–0.837)	0.941 (0.916–0.962)

*FNN* feedforward neural networks, *GNB* Gaussian naïve Bayes, *MLP* multi-layer perceptron, *LR* logistic regression, *SVM* support vector machine, *RF* random forest.

We further evaluated diagnostic performance across varying probability scores for renin-independent and renin-dependent SVM, FNN, and MLP models (Supplementary Fig. 3). This analysis demonstrated that compared to renin-dependent models, renin-independent models consistently exhibited narrower gray zone intervals between 95% sensitivity and 95% specificity thresholds.

### Diagnostic performance of ML models and the ARR

**Fig. 4:**
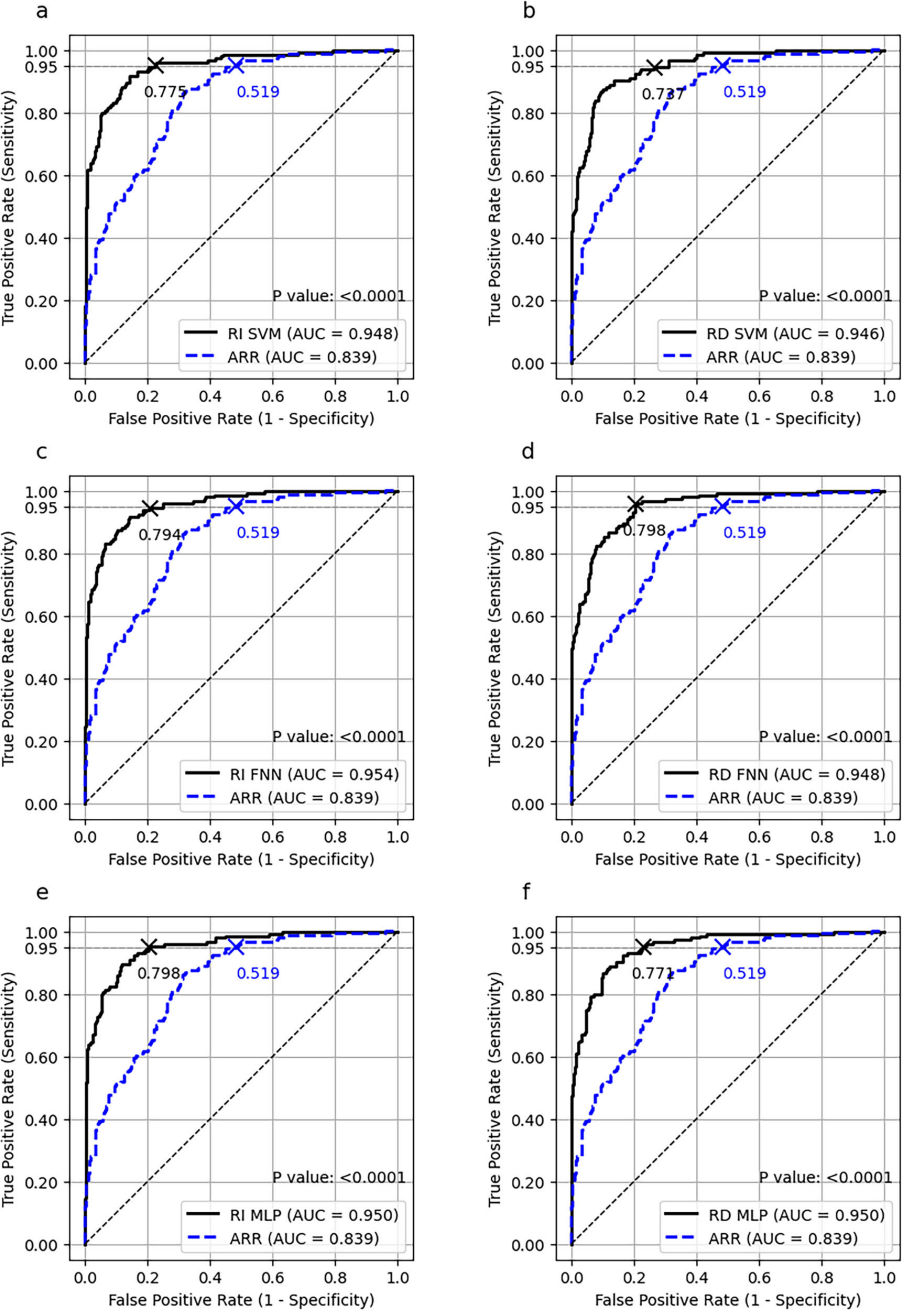
Comparison of machine learning models and the aldosterone:renin ratio. Panels show receiver operating characteristic (ROC) curve comparisons of the aldosterone:renin ratio (ARR) with the best performing renin-independent (RI) and renin-dependent (RD) machine learning (ML) models developed and validated using the combined SPISCA and PROSALDO cohorts. Comparisons are shown for support vector machine (SVM) (**a**, **b**), feedforward neural network (FNN) (**c**, **d**), and multilayer perceptron (MLP) (**e**, **f**) renin-independent (**a**, **c**, **e**) and renin-dependent (**b**, **d**, **f**) models. Black solid lines represent ML models, and blue dashed lines represent the ARR. Cross markers, together with the annotated values, indicate the specificity achieved at a predefined optimal sensitivity of 95%. Areas under ROC curves (AUCs) are provided for each model and ARR. *P* values indicate pairwise comparisons between ML models and the ARR.

Final comparisons of the renin-independent and renin-dependent FNN, MLP, and SVM models with the ARR demonstrated superior (*P* < 0.0001) performance of all six ML models compared to the ARR (Fig. 4). Diagnostic specificity and rates of false-positives for each model and the ARR were established according to optimal sensitivities between 85% to 95% from examination of ROC curve tables (Supplementary Tables 5 and 6). At cut-offs of the ARR of 21.0 and 26.6 pmol/mU to allow for diagnostic sensitivities of 95% and 90%, respectively, specificities were 52% and 61%. In contrast, for the renin-independent SVM, FNN and MLP models, cut-offs for probability scores to achieve 95% and 90% sensitivity were associated with respective specificities of 78% to 80% and 87% to 89% (Fig. 4). At those respective sensitivities of 95% and 90%, and under the composite reference standard framework of the study, the higher specificities for ML models than the ARR were associated with 53% to 58% and 67% to 72% respective reductions in false-positive results for probability scores compared to the ARR.

**Fig. 5:**
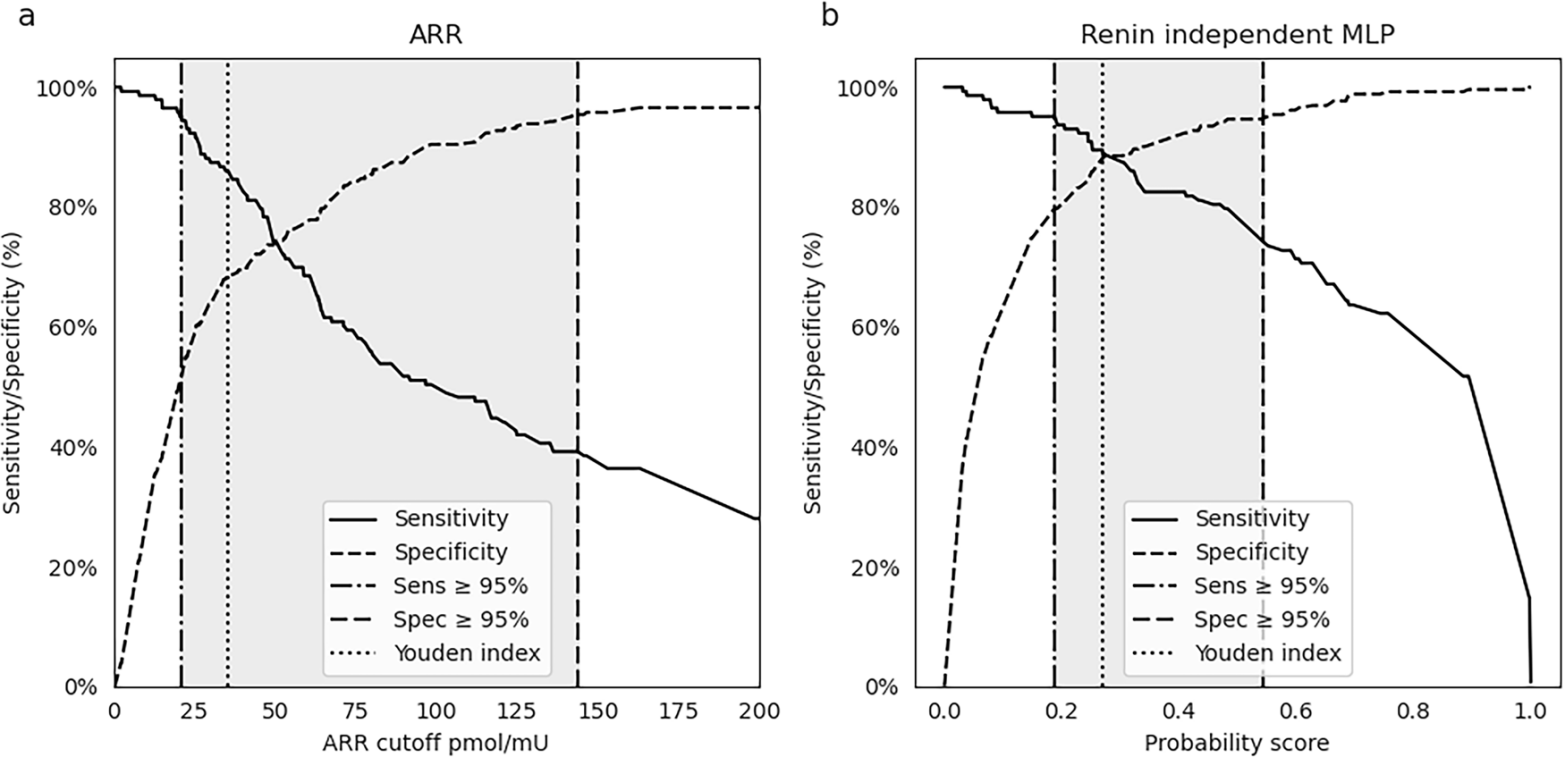
Threshold-dependent diagnostic performance of the aldosterone:renin ratio and renin-independent multilayer perceptron model. Panels show sensitivity and specificity analysis across varying decision thresholds for aldosterone:renin ratio (ARR) (**a**) and the renin-independent multilayer perceptron (MLP) model (**b**). Sensitivity (solid lines) and specificity (dashed lines) are shown across decision thresholds. Vertical lines denote the Youden-optimal threshold and those corresponding to 95% sensitivity and 95% specificity. The interval between the 95% sensitivity and 95% specificity thresholds is shown as the gray zone.

Additional sensitivity and specificity analyses across varying cut-offs for the ARR and probability scores for ML model (Fig. 5) showed that the renin-independent MLP model maintained favorable diagnostic performance across a broad range of probability scores and exhibited a narrower gray zone than the ARR, defined by the interval between the 95% sensitivity and 95% specificity thresholds.

**Fig. 6:**
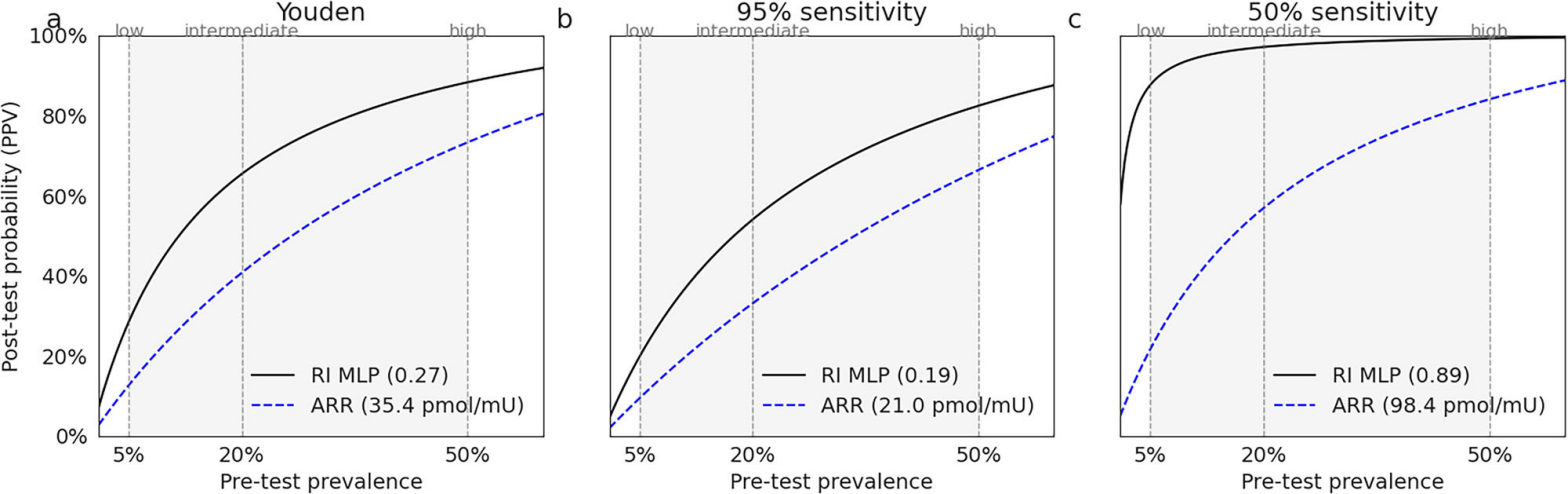
Post-test probability across varying pre-test prevalence. Panels show post-test probability as a function of pre-test prevalence for the renin-independent (RI) multilayer perceptron (MLP) model and aldosterone:renin ratio (ARR) using the Youden index-based threshold (**a**), the threshold corresponding to 95% sensitivity (**b**), and the threshold corresponding to 50% sensitivity (**c**). Performance is illustrated across clinically relevant pre-test probabilities (5%, 20%, and 50%), representing low-, intermediate-, and high-probability settings, respectively.

We then translated the above findings into clinically relevant screening contexts by evaluating post-test probabilities across different pre-test prevalences of PA, which showed that all renin-independent and renin-dependent ML models consistently yielded higher post-test probabilities and fewer false-positive results per 1000 screened patients than the ARR (Supplementary Fig. 4 and Supplementary Table 7). As further illustrated in Fig. 6, the renin-independent MLP model yielded higher post-test probabilities than the ARR across the full range of clinically relevant pretest prevalences and according to different cut-offs, including those calculated from Youden’s index, as well as at lower cut-offs to achieve 95% sensitivity and higher cut-offs to achieve high positive predictive value.

## Discussion

This study establishes plasma steroidomics-based ML models for screening patients with suspected PA that at all levels of prevalence considerably improve upon previous models by incorporation of serum potassium and renin. Among those two additional features, potassium provides a better boost to diagnostic performance than renin. Both renin-independent and renin-dependent models demonstrate similarly superior diagnostic performance compared to conventional use of the ARR. Minimized need for antihypertensive medication washout with renin-independent models, however, provides an advantage over renin-dependent models to avoid adverse events and improve patient safety.

Three studies related to the use of ML for screening for PA have been described previously^15–17^. In key metrics such as AUC, sensitivity, and specificity, our ML models outperformed the first two for screening^15,16^. In the third study, Reel et al.^17^ described an ML model with an AUC of 0.95, similar to the models of the present study. However, external validation was limited, and the model employed 46 selected features, whereas comparable performance of our models with sex- and age-adjustments for steroids was achieved with six or fewer features. The simplified feature set reduces the need for extensive and potentially costly laboratory tests and thus provides a more pragmatic approach for routine clinical practice.

With the above considerations in mind, two other centers (in Australia and the United Kingdom) have already established the required mass spectrometry-based steroidomics and are applying this together with previously established ML models for diagnostic stratification of PA. The presently developed models only provide for the first improved step in that process. Further ML models using the same data derived from screening samples are then applied to facilitate stratification of patients for therapeutic intervention, a process that promises to minimize need for confirmatory and subtyping tests^18,19^. As we have shown in collaboration with one of the two aforementioned centers, the technology offers strong robustness according to reproducibility of ML model outputs within and between laboratories that exceeded reproducibility of underlying steroid measurements^20^. Such generalizability is critical for harmonized international deployment, which becomes particularly important under new guidelines that call for expanded screening for PA among hypertensive populations^21^. With false-positive rates for the ARR of 39% to 48% at sensitivities optimal for screening, it can be appreciated that such expanded screening would increase pressure for specialist confirmatory and subtyping tests.

Under the reference standard framework of this study, the 53–72% reductions in false-positive results for ML models compared to the ARR illustrate minimized need for subsequent confirmatory and subtyping diagnostic procedures. Furthermore, this can be achieved using renin-independent models that minimize need for antihypertensive medication washout. Wider translation into clinical practice beyond current centers with the technology will, however, take time. ML models should ideally also be integrated within clinical decision support systems. The currently described models comprise only the first step for a single sample test to minimize need for confirmatory and subtyping tests^22^. In the interim, renin-dependent models, with no or minimal need for steroid profiling, might also be developed for screening.

The primary limitation of this study, as in other diagnostic studies of PA, concerns imperfect accuracy of any single reference standard to unequivocally confirm and exclude patients with suspected PA who do not undergo adrenalectomy. Therefore, rather than a single reference standard for those patients, we used a combination of reference standards. The most important was the seated saline suppression test (SSST) with liquid chromatography-tandem mass spectrometry (LC–MS/MS) measurements of aldosterone, used to exclude or confirm PA among patients with positive screening tests (i.e., either or both a positive ARR or ML-probability score). Although there has been some confusion about use and validity of the SSST, this has been addressed in a commentary by members of the 2025 Endocrine Society (ES) guideline committee on PA to clarify that the SSST remains a valuable confirmatory test as long as standard operating procedures are correctly followed^23^. As we have validated separately^24^, at recommended cut-offs of 162 pmol/L the test offers high sensitivity so that few patients with PA are missed. That small proportion of misclassified cases may have deflated accuracy of the ARR relative to ML models. However, the more important limitation of the SSST at the cut-off of 162 pmol/L is false-positive results that may inflate accuracy of the ARR relative to ML models. To partially offset the above and other limitations, outcome assessments were used to further confirm or exclude PA in 358 of the 825 patients in whom diagnosis of PA was not possible based on gold standard PA surgical outcome (PASO)^25^ or HISTALDO (histopathology of primary aldosteronism) criteria^26^.

Although these additional outcome assessments performed more than a year after initial screening provide a means to correct or confirm classifications based on earlier tests, it remains possible that some patients with early-stage disease might have been missed. Use of a normal ARR at outcome assessment to further exclude PA (or, without that, both a normal ARR and steroid profile at screening) might also introduce incorporation bias and inflate diagnostic accuracy of the ARR and ML models, though more so the former. Finally, exclusion of 79 patients in whom disease classifications could not be achieved may have introduced some degree of selection bias.

A limitation specific to examination of prevalence-post-test probability relationships is that diagnostic performance was not explicitly evaluated within clinically defined subgroups, such as patients with only hypertension versus others at higher risk of PA. Prevalence-based estimates of positive predictive value were derived from the overall cohort and may therefore not fully capture variation across different clinical settings or levels of pre-test suspicion. Accordingly, post-test probability estimates may be affected by spectrum bias and should be interpreted with caution.

Despite the above limitations, the present study also offers several strengths compared to other studies. Primary strengths include the large sample size, availability of all features, and accurate measurements of all steroids by LC–MS/MS. Furthermore, patient management was closely guided in the PROSALDO trial by advanced technologies, including digital data collection platforms, a prototype clinical decision support system, and the prospective design, which minimizes bias. Importantly, and as detailed in the methods and supplement, classification of patients with and without PA was achieved by rigorously applied criteria, and the ML training process was conducted in accordance with the TRIPOD-AI guideline^27^, essential for robust model development and transparent reporting. Critical aspects included care with identification of candidate features, data preparation, the fit of prediction models with appropriate datasets, consideration of overfitting through regularization techniques, and performance of both internal and external validation to assess model performance. Adherence to the above procedures enhances the reliability and clinical applicability of the developed models.

To summarize, our study highlights the substantial improvements achieved with the new ML models over conventional use of the ARR for screening and the potential of MLP, FNN, and SVM models in classification tasks. Improved screening using these ML models represents one step that, from a single blood sample, will also allow for more effective and efficient subtype classification than currently possible. The consistent performance enhancements across different datasets affirm the reliability and effectiveness of ML approaches, paving the way for a reliable, robust, and efficient clinical decision support system for diagnostic stratification of PA.

## Methods

This study was reported in accordance with the TRIPOD-AI^27^ (Transparent Reporting of a multivariable prediction model for Individual Prognosis Or Diagnosis—Artificial Intelligence extension) checklist. The full completed checklist is provided with the Supplementary Material (Supplementary Table 8).

### Patient datasets and recruitment

Data from the SPISCA cohort were sourced from previously established datasets^18^. Recruitment of patients into the PROSALDO trial (registration no: DRKS00017084) was based on criteria also employed for patients with suspected PA of the SPISCA study: an office blood pressure >150/100 mmHg, therapy-resistant hypertension or hypertension associated with hypokalemia, hemorrhagic stroke, an adrenal incidentaloma, or obstructive sleep apnea. Exclusion criteria included presence of other forms of secondary hypertension, necessity for continued use of medications that prevented interpretation of laboratory test results, pregnancy, comorbidities, and other conditions that precluded required investigational procedures and/or therapeutic interventions, and impaired mental capacity that precluded informed consent.

Patients were recruited into the PROSALDO trial between February 2019 and July 2025, with recruitment into the first phase completed in December 2023 and the second phase in July 2025. The study was conducted in accordance with the principles of the Declaration of Helsinki, and all patients provided written informed consent. The study protocol was approved by the local ethics committees at the six participating tertiary care centers: University Hospital Carl Gustav Carus, Technische Universität Dresden, Germany (IRB no. EK 386102018); University Hospital Würzburg, Germany (IRB no. 42/19); University Hospital Ludwig-Maximilian Munich, Germany (IRB no. 18-117); University of Zurich, Switzerland (IRB no. 2018-01292); Hudson Institute of Medical Research, Clayton, Australia (IRB no. RES-19-0000480A); and Prince of Wales and St George Hospitals, Sydney, Australia (2020/PID03277, IRB no. RES-19-0000480A).

### Disease classification

Confirmation and exclusion of PA for purposes of classification generally followed procedures outlined in the 2016 and 2025 ES guidelines^21,28^. In both the SPISCA study and PROSALDO trial, routine local measurements of serum potassium and plasma aldosterone and renin were followed by centralized LC–MS/MS measurements of plasma steroids from the same blood specimens used for local routine measurements of aldosterone and renin.

Blood samples for routine measurements of aldosterone and renin and LC–MS/MS-measured steroid profiles were collected in the morning hours and in the seated position. Patients with hypokalemia received potassium supplementation as necessary. Screening was generally carried out after washout of antihypertensive medications known to impact the renin-angiotensin-aldosterone system. For the PROSALDO trial, there were two phases for screening, such that for some patients, screening was first carried out without medication washout. For those patients, a second screen with medication washout and adjustment to antihypertensive medications without impact on the renin-angiotensin-aldosterone system (e.g., alpha-adrenoceptor blockers, non-dihydropyridines calcium channel blockers, vasodilators) was required. This was particularly important if the results of initial screening were rendered non-interpretable by use of particular classes of antihypertensive medications (e.g., a normal ARR in patients taking angiotensin receptor antagonists or angiotensin converting enzyme inhibitors).

Screening with measurements of aldosterone and renin was followed in patients with positive test results by confirmatory tests, which for most patients involved the SSST. Similar to screening, the SSST was stipulated to be carried out after washout of interfering medications, correction of hypokalemia, and in the morning hours. As outlined elsewhere^24^, one difference between procedures of the SPISCA study and the PROSALDO trial involved use in the latter of either positive results of the ARR or ML-derived steroid probability scores to guide patients to the SSST. Adrenal venous sampling and/or imaging of the adrenals was reserved for patients with positive results for confirmatory tests who were also willing to undergo adrenalectomy according to results of subtyping studies. For patients who underwent adrenalectomy, outcomes were based largely on the PASO criteria^25^.

Among the 212 patients with PA from the SPISCA study, for 139 the diagnosis was based on biochemical cure after adrenalectomy according to PASO criteria^25^. For the other 73, the diagnosis was based on positive confirmatory test results. For the 140 patients without PA, this classification was based on negative results of the ARR or negative results of confirmatory tests following positive screening test results.

Among all 949 patients of the PROSALDO trial in whom classifications were possible, there were 115 in whom PA was confirmed by biochemical cure after adrenalectomy, according to PASO criteria^25^. For 104 of those patients, further confirmation was also based on histopathological findings of aldosterone synthase-positive adrenal lesions according to HISTALDO criteria^26^. For another nine patients who underwent adrenalectomy, and who were either not cured (*n* = 4) or who did not make it to outcome assessment, PA was confirmed by HISTALDO criteria alone. Thus, among all 254 PROSALDO patients classified with PA, the diagnosis was confirmed in 124 by either or both PASO or HISTALDO criteria. Among the other 130 patients, PA was confirmed in 46 by positive results of screening and confirmatory tests, followed by continuing positive biochemical test results at outcome assessment ≥12 months after the last procedure. Confirmation of PA at outcome assessment was not possible in 22 patients due to use of interfering medications, so that for these and another 61 classifications of PA depended on both positive results at screening and a positive SSST according to LC–MS/MS measurements of aldosterone.

PA was considered excluded in 312 of 695 PROSALDO patients by continued negative biochemical test results at outcome assessment that followed negative screening or confirmatory test results. For another 212 patients, negative results of the SSST were used to classify patients without PA. Exclusion of PA in the 171 other patients was based on negative screening results (*n* = 119) and/or LC–MS/MS-measured seated morning plasma aldosterone concentrations spontaneously below 162 pmol/L (*n* = 99), the cut-off defined for the SSST in the 2025 ES guideline^21^. A cut-off of 162 pmol/L for LC–MS/MS measured aldosterone was used to define positive versus negative results for the SSST, as developed by Stowasser et al and further validated by us^23,24,29,30^.

As described previously^5^, cut-offs for the ARR were dependent on local practice and the assay method, as also currently recommended under the 2025 ES guideline^21^. For purposes of screening, most centers used chemiluminescence immunoassays (CLIA) to measure plasma aldosterone and renin. At European centers, which accounted for over 90% of all patients enrolled into protocols, cut-offs for the ARR were between 31 and 33 pmol/mU, which ensures high diagnostic sensitivity for screening. For Australian centers, cut-offs were in accordance with the 2016 ES guideline^28^: 70 pmol/mU for one center that employed the Liaison CLIA and 50 pmol/mU for the other that used LC–MS/MS for aldosterone and the Liaison CLIA for plasma renin.

### Steroid profiles

Measurements of plasma steroids utilized a previously established LC–MS/MS method^31^. Steroids considered as features for ML models included aldosterone, 18-oxocortisol, 18-hydroxycortisol, 11-deoxycortisol, 11-deoxycorticosterone, cortisol, cortisone, corticosterone, 21-deoxycortisol, 17-hydroxyprogesterone, androstenedione, dehydroepiandrosterone (DHEA) and DHEA-sulfate (DHEAS).

### Model development

In accordance with the TRIPOD-AI guideline^27^, the supervised ML workflow comprised several phases (Fig. 1a): data analysis and preprocessing, feature selection, model training, internal testing, external validation, with supplementary evaluations that included probability calibration (Supplemental Fig. 5) and concordance assessment (Supplemental Fig. 6).

The data preprocessing pipeline consisted of several critical steps, including data cleaning, integration, confounder adjustment, and normalization. No missing values were present in measurements; therefore, no imputation was required. Samples with indeterminate classification were removed based on assessments by clinical experts, as described above. Data integration was performed for the PROSALDO cohort using data from two screening and one confirmatory test phases. Data were selected and consolidated by averaging values for two or more samples collected after medication washout.

For both SPISCA and PROSALDO cohorts, steroid measurements underwent confounder adjustments using reference-population-based normalization for age and sex as outlined previously^18,32^. To account for differences in feature scale and distribution, four normalization strategies were evaluated, including standard scaling, robust scaling, min–max normalization, and quantile transformation. Features were selected based on statistical scoring (SelectKBest), model feature importance (SelectFromModel), or recursive feature elimination (RFE)^33^. SelectKBest was used to select features based on statistical tests, while RFE iteratively removed the least important features to identify the most relevant subset. SelectFromModel was used as a wrapper-based feature selection method that employed feature importance scores derived from a trained RF model, computed as the mean decrease in Gini impurity. The number of selected steroids was not fixed a priori and was evaluated across a range of three to ten features to identify the optimal number for each model. PI values were used to assess the contribution of individual features for predictions of models.

ML models were developed using six different ML algorithms: SVM, FNN, MLP, LR, RF, and GNB. To account for moderate class imbalance in the development cohort, class weights were applied during training for most models, where supported by the underlying algorithm. No resampling techniques were applied, and calibration was assessed on the test set. Hyperparameters were optimized using Bayesian optimization within a fivefold cross-validation framework, with feature selection and normalization performed within each cross-validation fold. The ML pipeline iteratively evaluated four normalization strategies and three feature selection methods across all combinations.

After model training, we applied 500 bootstrap resampling iterations for internal testing and external validation and compared performance metrics according to balanced accuracy, AUCs, F1 scores, sensitivity, specificity, and Matthews correlation coefficient. Since the ML models were developed for purposes of screening, we considered a sensitivity of 95% and 90% as optimal to identify models with highest specificity. In addition, to support clinically relevant interpretation, we evaluated positive predictive probabilities across a range of pre-test prevalences based on model-derived sensitivity and specificity. All analyses were performed in Python. ML models were implemented primarily using scikit-learn^33^, with neural network models constructed using the Keras API with TensorFlow^34^ as backend.

### Statistical analysis

Continuous parameters are shown as geometric means with 95% confidence intervals (CI). Statistical tests included the Mann–Whitney U, multivariable linear regression, and Chi-square tests, which were conducted using SciPy^35^ and Statsmodels^36^. The algorithm for paired AUCs as described by DeLong et al.^37,38^ was used to evaluate differences in AUCs. Optimal sensitivities and specificities were calculated using Youden’s index. Sample size was based on a power calculation for the primary objective of the PROSALDO protocol, as described in the clinical protocol for this trial (trial registration no: DRKS00017084 https://drks.de/search/en/trial/DRKS00017084; registered on 17 January 2020).

## Supplementary information

Supplementary appendix

## Data Availability

The datasets generated and/or analyzed during the current study are not publicly available due to patient privacy and institutional ethical restrictions, but are available from the corresponding author on reasonable request.

## Code availability

The code used for the analyses in this study is available from the corresponding author on reasonable request.
