# Supplementary material for: Advanced machine learning-based screening for primary aldosteronism with plasma steroids, potassium, and renin
Source: NPJ Digit Med. 2026 Jun 24;9:484. doi: 10.1038/s41746-026-02906-w (PMC13294501; doi:10.1038/s41746-026-02906-w)
Supplement: Supplementary file 1 — Supplementary appendix [file 41746_2026_2906_MOESM1_ESM.pdf]

Supplemental Appendix

Advanced Machine Learning-Based Screening for Primary Aldosteronism with Plasma Steroids, Potassium, and Renin

Wenyu Zhang,[1] Christina Pamporaki,[2] René Jäkel,[1,3] Georgiana Constantinescu,[2] Mirko Peitzsch,[4] Manuel Schulze,[3] Jun Yang,[5,6] Andrea Rita Horvath,[7] Ralph Müller-Pfefferkorn,[3] Tracy Ann Williams,[7] Martin Reincke,[7] Felix Beuschlein,[7,8] Carmina Teresa Fuss,[9] Stefanie Hahner,[9] Graeme Eisenhofer[2]

[1]Center for Scalable Data Analytics and Artificial Intelligence Dresden/Leipzig, Technische Universität Dresden, Germany; [2]Department of Medicine III, University Hospital Carl Gustav Carus, Technische Universität Dresden, Germany; [3]Center for Information Services and High Performance Computing, Technische Universität Dresden, Dresden, Germany; [4]Institute of Clinical Chemistry and Laboratory Medicine, University Hospital Carl Gustav Carus, Technische Universität Dresden, Germany; [5]Centre for Endocrinology and Reproductive Health, Hudson Institute of Medical Research and Department of Medicine, Monash University, Clayton, Australia; [6]New South Wales Health Pathology, Prince of Wales Hospital, Sydney, Australia; [7]Department of Medicine IV, University Hospital, Ludwig Maximilian University Munich, Munich, Germany; [8]Department of Endocrinology, Diabetology and Clinical Nutrition, University Hospital Zurich and University of Zurich and the LOOP Zurich Medical Research Center, Zurich, Switzerland; [9]Department of Internal Medicine I, Division of Endocrinology and Diabetes, University Hospital, University of Würzburg, Würzburg, Germany

Table of contents **Page**

Supplemental Results………………………………………………………………………... 2
- Characteristics and biochemical test results…………………………………………… 2
  - Supplementary Table 1…………………………………………………………… 2
- Development of ML models using the SPISCA dataset………………………………… 3
  - Supplementary Table 2…………………………………………………………… 3
  - Supplementary Figure 1…………………………………………………………… 4
- Impact of antihypertensive medications……………………………………………… 5
  - Supplementary Table 3…………………………………………………………… 5
- Training and testing on combined SPISCA and PROSALDO I cohorts………………… 6
  - Supplementary Figure 2…………………………………………………………… 6
  - Supplementary Table 4…………………………………………………………… 7
- Diagnostic performance across varying thresholds ……………………………………...8
  - Supplementary Figure 3……………………………………………………………..8
- Receiver operating characteristic (ROC) curve tables………………………………… 8
  - Supplementary Table 5…………………………………………………………… 9
  - Supplementary Table 6…………………………………………………………… 9
- Post-test probability across pre-test prevalence and decision thresholds……………….10
  - Supplementary Figure 4……………………………………………………………..10
  - Supplementary Table 7……………………………………………………………..11
- Model calibration……………………………………………………………………….11
  - Supplementary Figure 5…………………………………………………………… 12
- Model concordance..12
  - Supplementary Figure 6………………………………………………………… 13

TRIPOD-AI Checklist………………………………………………………………… 14
  - Supplementary Table 8…………………………………………………………… 14

Supplemental Results

Characteristics and biochemical test results

No significant differences were observed in age between patients with and without primary aldosteronism (PA) of both SPISCA and PROSALDO cohorts (Supplementary Table 1). A significant difference in sex distribution was observed between patients with and without PA of the PROSALDO cohort but not the SPISCA cohort. Serum potassium and renin concentrations were significantly lower in patients with PA compared to those without PA in both SPISCA and PROSALDO cohorts. Most steroids were higher in patients with than without PA in both SPISCA and PROSALDO cohorts. In contrast plasma concentrations of DHEAS were significantly lower in patients with than without PA in PROSALDO cohort. Plasma concentrations of cortisol did not differ between patients with and without PA for both cohorts, whereas cortisone was significantly lower in patients with than without PA for the SPISCA cohort but not the PROSALDO cohort.

Supplementary Table 1: Characteristics and biochemical test results for SPISCA and PROSALDO cohorts for comparing patients with and without PA.

	SPISCA			PROSALDO		
	PA	No PA	P-value	PA	No PA	P-value
Patient demographics						
N	212	140		192	578	
Sex (F/M)	92/125	72/68	0.0949	80/112	320/258	0.0010
Age (years)	49.8 (48.2-51.4)	44.6 (42-47.3)	0.0138	48.8 (47.2-50.5)	47.1 (46-48.2)	0.2877
Routine measurements						
Potassium (mmol/L)	3.31 (3.22-3.4)	4.03 (3.96-4.1)	<0.0001	3.62 (3.55-3.69)	4.19 (4.15-4.22)	<0.0001
Renin (mU/L)	3.76 (3.37-4.2)	12.12 (10.02-14.66)	<0.0001	4.62 (4.06-5.25)	11.01 (10.08-12.03)	<0.0001
LC-MS/MS Steroids (ng/mL)						
Aldosterone	0.183 (0.166-0.203)	0.063 (0.056-0.072)	<0.0001	0.200 (0.184-0.218)	0.071 (0.067-0.075)	<0.0001
18-Oxocortisol	0.042 (0.034-0.051)	0.014 (0.012-0.015)	<0.0001	0.053 (0.045-0.064)	0.015 (0.014-0.016)	<0.0001
18-Hydroxycortisol	1.101 (0.974-1.244)	0.617 (0.561-0.678)	<0.0001	1.124 (1.009-1.253)	0.623 (0.599-0.648)	<0.0001
11-Deoxycorticosterone	0.086 (0.075-0.099)	0.039 (0.034-0.044)	<0.0001	0.057 (0.051-0.064)	0.030 (0.028-0.031)	<0.0001
Corticosterone	3.01 (2.63-3.45)	2.33 (2.02-2.69)	0.0081	2.25 (2.03-2.5)	1.87 (1.76-1.98)	0.0011
Cortisol	121 (113-130)	132 (122-142)	0.1619	104 (99-110)	101 (98-104)	0.2462
Cortisone	17.3 (16.3-18.3)	19.8 (18.9-20.8)	0.0053	17.7 (17.0-18.4)	17.8 (17.5-18.2)	0.4599
11-Deoxycortisol	0.419 (0.373-0.472)	0.228 (0.2-0.26)	<0.0001	0.337 (0.305-0.371)	0.224 (0.212-0.226)	<0.0001
21-Deoxycortisol	0.022 (0.018-0.027)	0.012 (0.009-0.016)	0.0005	0.024 (0.022-0.027)	0.019 (0.018-0.020)	0.0002
17-Hydroxyprogesterone	0.859 (0.772-0.956)	0.542 (0.468-0.628)	<0.0001	0.590 (0.533-0.653)	0.431 (0.402-0.462)	0.0009
Androstenedione	1.214 (1.124-1.31)	1.075 (0.976-1.184)	0.0006	0.666 (0.622-0.714)	0.607 (0.582-0.632)	0.0685
DHEA	1.92 (1.71-2.15)	2.16 (1.868-2.5)	0.6842	2.46 (2.23-2.71)	2.43 (2.29-2.58)	0.8924
DHEAS	968 (882-1062)	1168 (1021-1336)	0.3376	925 (833-1026)	972 (910-1039)	0.0487

Continuous parameters are shown as geometric means with confidence intervals.

Development of ML models using the SPISCA dataset

Across steroid-only machine learning (ML) models, the normalization and feature selection methods varied. The selected feature sets overlapped across algorithms but were not identical. (Supplementary Table 2). Aldosterone, 18-oxocortisol and 11-deoxycorticosterone were included across all models. Other steroids (e.g.,18-hydroxycortisol, cortisol, cortisone and 21-deoxycortisol) were selected only in some models, resulting in final feature sets that ranged from three to seven steroids depending on the algorithm and feature-selection method. This was in line with the results of permutation importance (PI) analyses (Supplementary Figure 1, Panel a–f), where aldosterone consistently showed the highest importance, 18-oxocortisol ranked next, and 11-deoxycorticosterone showed a smaller contribution, whereas the remaining steroids generally showed low or near-zero importance.

Supplementary Table 2: Normalization and feature selection methods for each ML model, with corresponding steroid features. Models were trained on the SPISCA cohort and validated on the PROSALDO cohort.

	Model	Normalization	Feature selection	Selected Features	Steroid Number
Previous Model	SVM	LOG-FM*	RFE	ALD 18OXOF 18OHF DOC E S AE	7
Steroid	SVM	Min-max	RFE	ALD 18OXOF DOC 18OHF CORT 21DF	6
	FNN	Standard	RFE	ALD 18OXOF DOC 18OHF CORT 21DF	6
	MLP	Min-max	SKB	ALD 18OXOF18OHF 17-OHP S DOC	6
	LR	Min-max	RFE	ALD E 18OXOF DOC F E	5
	RF	Quantile	SFM	ALD 18OXOF 18OHF DOC	4
	GNB	Min-max	SFM	ALD 18OXOF DOC	3
Steroid +Potassium	SVM	Min-max	SFM	ALD 18OXOF K DOC	3
	FNN	Min-max	SKB	ALD 18OXOF K DOC	3
	MLP	Min-max	SFM	ALD 18OXOF K DOC	3
	LR	Min-max	SFM	ALD K DOC 18OXOF	3
	RF	Standard	SFM	ALD 18OXOF K DOC	3
	GNB	Robust	SFM	ALD 18OXOF K DOC	3
Steroid +Renin +Potassium	SVM	Min-max	RFE	ALD Renin K 18OXOF S	3
	FNN	Min-max	RFE	ALD Renin 18OXOF K	2
	MLP	Standard	SKB	ALD Renin K 18OXOF 18OHF DOC S 17-OHP	6
	LR	Min-max	RFE	ALD Renin K F S 18OXOF	4
	RF	Quantile	SKB	ALD Renin 18OXOF K DOC	3
	GNB	Quantile	SFM	ALD Renin K 18OXOF	2

FNN: feedforward neural networks; GNB: Gaussian naïve Bayes; MLP: multi-layer perceptron; LR: logistic regression; SVM: support vector machine; RF: random forest. Standard: standardization; Min-max: min-max scaling; Robust: robust scaling; SKB: SelectKBest; RFE: recursive feature elimination; SFM: SelectFromModel. ALD: aldosterone; 18OXOF: 18-oxo-cortisol; 18OHF: 18-hydroxycortisol; 21DF: 21-deoxycortisol; CORT: corticosterone; E: cortisone; DHEA: dehydroepiandrosterone; DHEAS: DHEA sulfate; DOC: 11-deoxycorticosterone; S: 11-deoxycortisol; 17-OHP: 17-hydroxyprogesterone; F: cortisol; AE: androstenedione; Potassium: K. *LOG-FM, logarithm to the base10 of the fold-difference of concentrations from an age and sex-specific reference population mean.

After the inclusion of potassium, most models selected min–max normalization, except for the random forest (RF) and Gaussian naïve Bayes (GNB) models (Supplementary Table 2). In addition, all models selected SelectFromModel (SFM), except the feedforward neural network (FNN) model combined with SelectKBest (SKB). The selected feature sets became smaller and potassium ranked highly in PI values followed by aldosterone and 18-oxocortisol for most models (Supplementary Figure 1). Correspondingly, potassium was the top contributor across models, with reduced importance for 11-deoxycorticosterone and other steroids (Supplementary Figure 1, Panel g–l); aldosterone and 18-oxocortisol remained the steroid features with the most apparent contributions.

With further inclusion of renin, the machine learning models selected different normalization and feature selection methods (Supplementary Table 2). The selected steroid subset was reduced again in many models. All models retained potassium and renin, but with only one or two steroid features (most often

aldosterone and/or 18-hydroxycortisol). Most other steroids were no longer selected except multilayer perceptron (MLP) and logistic regression (LR).

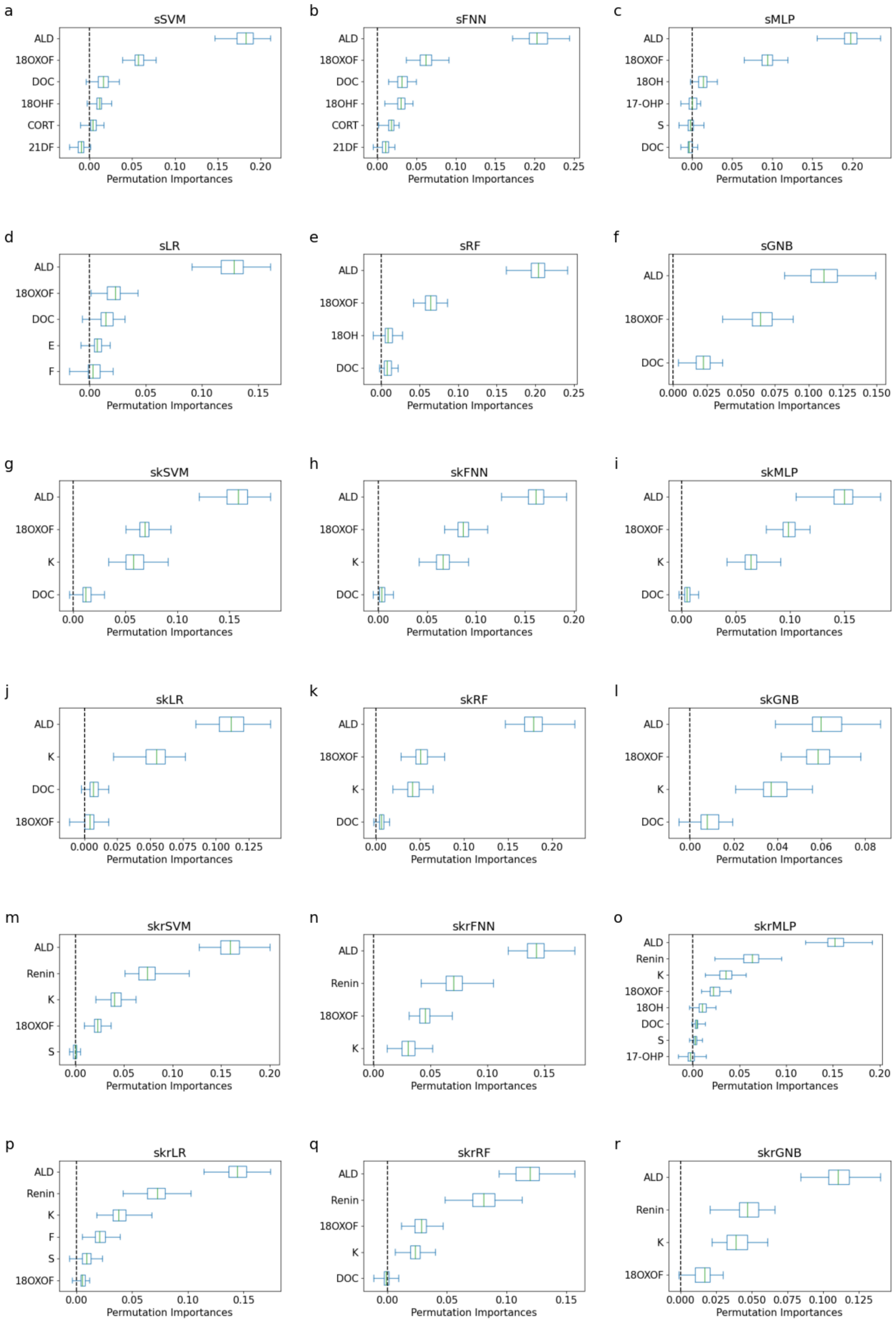

Supplementary Figure 1. Permutation importance (PI) box plot of selected features for SVM, FNN, MLP, LR, RF, and GNB models. Panel a-f: PI box plots for models trained only on the steroids. Panel g-l: PI box plots for models trained on the steroids and potassium. Panel m-r: PI box plots for models trained on steroids, potassium, and renin. *FNN: feedforward neural networks; GNB: Gaussian naïve Bayes; MLP: multi-layer perceptron; LR: logistic regression; SVM: support vector machine; RF: random forest. ALD: aldosterone; 18OXOF: 18-oxocortisol; 18OHF: 18-hydroxycortisol; 21DF: 21-deoxycortisol; CORT: corticosterone; E: cortisone; DHEA: dehydroepiandrosterone; DHEAS: DHEA sulfate; DOC: 11-deoxycorticosterone; S: 11-deoxycortisol; 17-OHP: 17-hydroxyprogesterone; F: cortisol; AE: androstenedione; Potassium: K.*

Impact of antihypertensive medications

According to available paired samples before and after medication washout, we selected 200 patients from the PROSALDO dataset to investigate impacts of antihypertensive medications, 55 with PA and 145 without PA (Supplementary Table 3). Antihypertensive medications included those expected to reduce renin (e.g., beta-adrenoceptor blockers) and others known to increase renin (e.g., angiotensin-converting enzyme inhibitors, angiotensin receptor blockers, diuretics). As part of medication washout, those medications known to influence the renin-angiotensin-aldosterone system were replaced by medications without influence on the renin-angiotensin-aldosterone system (e.g., alpha-adrenoceptor blockers and non-dihydropyridine calcium channel blockers). Due to use of multiple different classes of antihypertensive medications by many patients and the limited sample size of patient populations it was not possible in the present study to examine impacts of specific antihypertensive medications.

Supplementary Table 3: Characteristics and biochemical test results of the PROSALDO data subsets before and after medication washout.

	PA			No PA		
	Before washout	After washout	P-value	Before washout	After washout	P-value
Routine measurements						
Potassium (mmol/L)	3.56 (3.43-3.71)	3.59 (3.45-3.74)	0.8433	4.15 (4.08-4.21)	4.15 (4.08-4.22)	0.9514
Renin (mU/L)	8.25 (5.86-11.60)	6.61 (5.16-8.46)	0.5539	19.05 (14.54-25.0)	11.97 (9.99-14.36)	0.0588
LC-MS/MS Steroids (ng/mL)						
Aldosterone	0.180 (0.151-0.214)	0.210 (0.177-0.249)	0.2700	0.066 (0.058-0.074)	0.069 (0.062-0.077)	0.4206
18-Oxocortisol	0.058 (0.041-0.082)	0.055 (0.04-0.076)	0.8483	0.017 (0.015-0.019)	0.017 (0.015-0.019)	0.7245
18-Hydroxycortisol	1.094 (0.88-1.36)	1.209 (0.999-1.462)	0.4026	0.631 (0.583-0.682)	0.701 (0.646-0.76)	0.0526
11-Deoxycorticosterone	0.050 (0.041-0.061)	0.056 (0.044-0.069)	0.5360	0.030 (0.027-0.033)	0.031 (0.028-0.034)	0.8533
Corticosterone	1.848 (1.522-2.243)	2.344 (1.883-2.917)	0.1020	1.727 (1.524-1.959)	2.057 (1.812-2.335)	0.0461
Cortisol	104 (94-115)	110 (98-123)	0.1531	98 (91-104)	107 (100-115)	0.0446
Cortisone	18.5 (16.9-20.3)	17.2 (15.6-18.9)	0.1497	17.5 (16.7-18.3)	16.8 (16.1-17.6)	0.1558
11-Deoxycortisol	0.336 (0.266-0.425)	0.343 (0.278-0.424)	0.7721	0.240 (0.216-0.269)	0.241 (0.217-0.267)	0.9799
21-Deoxycortisol	0.023 (0.019-0.028)	0.030 (0.024-0.038)	0.0521	0.019 (0.016-0.021)	0.021 (0.019-0.024)	0.1910
17-Hydroxyprogesterone	0.553 (0.473-0.646)	0.563 (0.482-0.66)	0.7764	0.363 (0.315-0.418)	0.405 (0.354-0.464)	0.2750
Androstenedione	0.681 (0.619-0.749)	0.644 (0.582-0.713)	0.6219	0.569 (0.523-0.619)	0.549 (0.506-0.595)	0.5922
DHEA	2.42 (2.05-2.86)	2.34 (1.95-2.8)	0.9547	2.20 (1.96-2.47)	2.04 (1.81-2.30)	0.3697
DHEAS	1003 (827-1216)	917 (774-1090)	0.4300	875 (761-1006)	822 (716-944)	0.4794

Continuous parameters are shown as geometric means with confidence intervals.

The renin-independent models included FNN, support vector machine (SVM), MLP, RF, LR and GNB algorithms trained on steroids and potassium concentrations. The renin-dependent models included FNN, GNB, MLP, LR, RF, and SVM trained on steroids, potassium, and renin concentrations. In the selected subset, most biochemical measures were similar before and after medication washout in both patients with and without PA, including most steroids, potassium, and renin concentrations. Exceptions were cortisol and corticosterone in patients without PA. The absence of an overall effect on renin and aldosterone may reflect the use of antihypertensive medications before washout that have divergent effects on the renin–angiotensin–aldosterone system, as outlined above.

Training and testing on combined SPISCA and PROSALDO I cohorts

We combined data from the SPISCA and the first phase of PROSALDO datasets to maximize sample size and leverage as much available information as possible. We developed two sets of models: renin-independent models, trained using plasma steroid and serum potassium, and renin-dependent models that additionally incorporated plasma renin. These two model types were systematically compared to evaluate their respective screening performance.

Renin-independent models

For the renin-independent models, all except the RF model used min-max normalization. The RF model was the only model that applied a quantile transformation. Feature selection differed across models: recursive feature elimination (RFE) was combined by SVM, FNN, MLP, and LR models, while SKB was integrated with GNB and RF models. The number of selected steroid features ranged from three (for GNB and RF) to seven (for LR). Steroids such as aldosterone, 18-oxocortisol, and 11-deoxycorticosterone were consistently selected as important features with high PIs by most renin-independent models (except for LR) (Supplementary Figure 2, Panel a-f). Unlike other renin-independent models, the LR model incorporated a broader set of seven steroids for training.

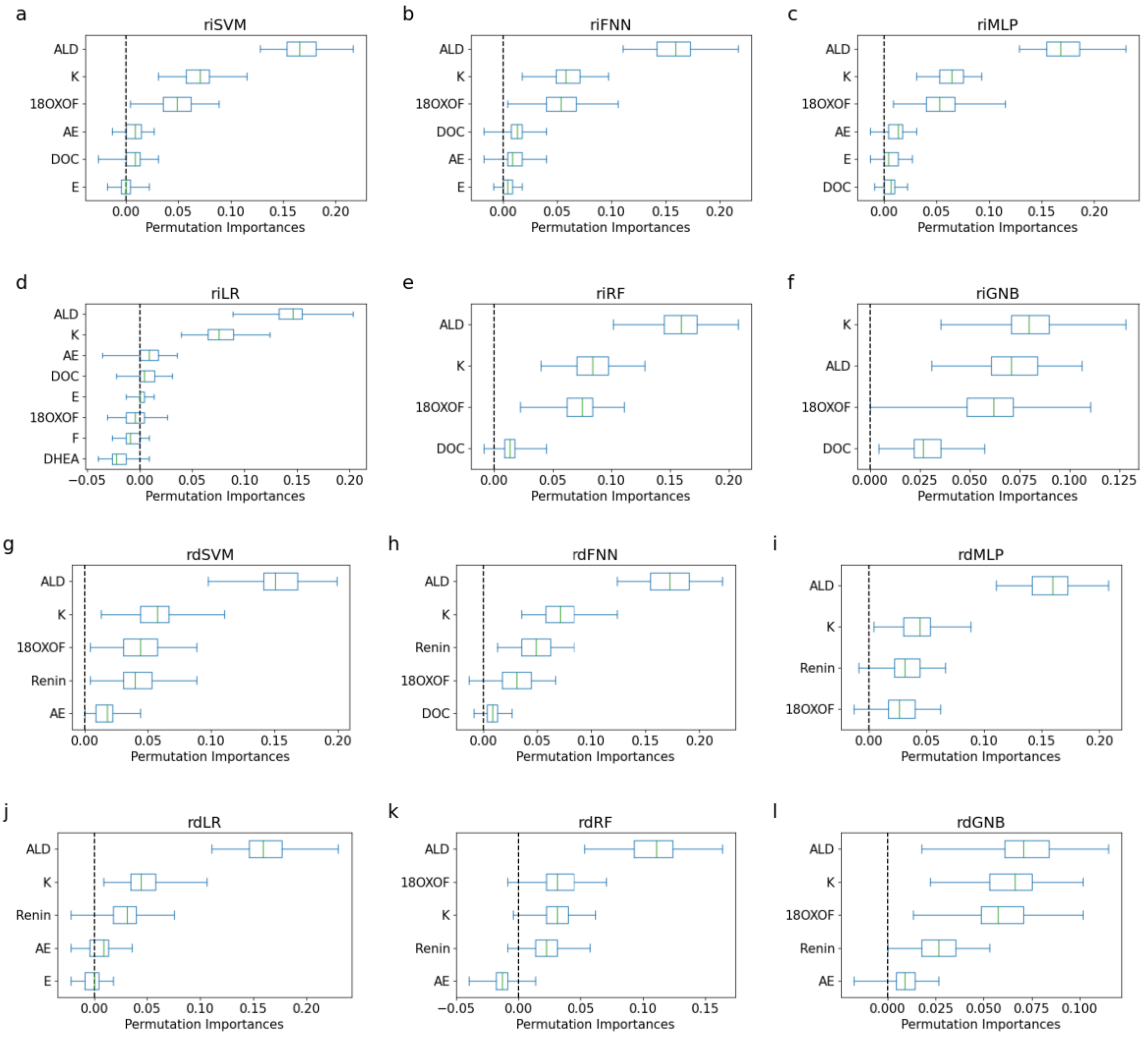

Supplementary Figure 2. PI box plots of selected features for SVM, FNN, GNB, MLP, LR, and RF models in patients without PA and with PA. The ML models were trained and tested on SPISCA and PROSALDO combined subsets. Panel a-f: PI box plots for renin-independent models. Panel g-l: PI box plots for renin-dependent models. FNN: feedforward neural networks; GNB: Gaussian naïve Bayes; MLP: multi-layer perceptron; LR: logistic regression; SVM: support vector machine; RF: random forest. ALD: aldosterone; 18OXOF: 18-oxocortisol; 18OHF: 18-hydroxycortisol; 21DF: 21-deoxycortisol; CORT: corticosterone; E: cortisone; DHEA: dehydroepiandrosterone; DHEAS: DHEA sulfate; DOC: 11-deoxycorticosterone; S: 11-deoxycortisol; 17-OHP: 17-hydroxyprogesterone; F: cortisol; AE: androstenedione; Potassium: K.

The six renin-independent models, SVM, FNN, MLP, LR, RF, and GNB, showed similar diagnostic performance for both internal and external validations with average AUCs of 0.943 and 0.949 (Supplementary Table 4). The renin-independent FNN model achieved the highest AUC, F1, ACC, and MCC in both internal and external validations. However, its performance did not markedly exceed that of the other models, including SVM, MLP, LR, and RF, all of which achieved AUCs above 0.939 in the internal validation and above 0.944 in the external validation. GNB showed the lowest ACC (0.857 and 0.872) and AUC (0.932 and 0.943) in both internal and external validations.

The renin-independent MLP, FNN, and SVM models were selected for further analysis on the basis of their superior overall performance among the candidate approaches. Among these, three renin-independent models could be ranked according to gray-zone width, with FNN showing the narrowest gray zone, followed by MLP, and then SVM (Supplementary Figure 3, Panel a,c,e). This indicates that FNN provided the most decisive separation between the sensitivity ≥95% and specificity ≥95% thresholds, indicating a smaller indeterminate range and more decisive separation between rule-out and rule-in classifications.

Supplementary Table 4: Evaluation metrics for classification performance of ML models trained on the SPISCA and PROSALDO combined cohort after the internal validation set.

Model	Balanced Accuracy	F1 score	MCC	AUC
		Renin-independent model		
SVM	0.877 (0.857-0.908)	0.831 (0.809-0.877)	0.731 (0.698-0.806)	0.944 (0.923-0.962)
FNN	0.893 (0.869-0.920)	0.857 (0.827-0.891)	0.774 (0.725-0.828)	0.951 (0.934-0.968)
MLP	0.886 (0.865-0.917)	0.840 (0.814-0.881)	0.746 (0.704-0.812)	0.947 (0.927-0.965)
LR	0.869 (0.846-0.902)	0.817 (0.796-0.874)	0.710 (0.676-0.811)	0.942 (0.922-0.960)
RF	0.867 (0.843-0.901)	0.826 (0.795-0.873)	0.726 (0.685-0.808)	0.939 (0.917-0.958)
GNB	0.857 (0.831-0.890)	0.821 (0.780-0.859)	0.727 (0.650-0.786)	0.932 (0.910-0.952)
		Renin-dependent model		
SVM	0.877 (0.849-0.910)	0.838 (0.801-0.878)	0.745 (0.683-0.809)	0.948 (0.930-0.966)
FNN	0.879 (0.854-0.909)	0.828 (0.801-0.876)	0.728 (0.686-0.804)	0.952 (0.934-0.968)
MLP	0.877 (0.855-0.913)	0.831 (0.804-0.875)	0.731 (0.687-0.805)	0.953 (0.935-0.969)
LR	0.867 (0.841-0.901)	0.818 (0.787-0.863)	0.709 (0.665-0.790)	0.947 (0.930-0.964)
RF	0.882 (0.857-0.912)	0.839 (0.809-0.883)	0.744 (0.697-0.817)	0.947 (0.928-0.964)
GNB	0.882 (0.857-0.910)	0.839 (0.807-0.874)	0.744 (0.691-0.801)	0.939 (0.915-0.959)

FNN: feedforward neural networks; GNB: Gaussian naïve Bayes; MLP: multi-layer perceptron; LR: logistic regression; SVM: support vector machine; RF: random forest.

Renin-dependent models

For the renin-dependent models, normalization strategies varied across models. In terms of feature selection, several models employed RFE, including GNB, LR, RF, and SVM models. The FNN model used SKB, while the MLP model applied SFM. Most renin-dependent models, including FNN, GNB, LR, RF, and SVM, selected three steroids during training. In contrast, the MLP model selected only two: aldosterone and 18-oxocortisol. Interestingly, potassium consistently exhibited higher PIs than renin across all renin-dependent models (Supplementary Figure 2, Panel g-l).

The six renin-dependent models (SVM, RF, GNB, FNN, LR, and MLP) showed broadly comparable diagnostic performance in both internal and external validations, with mean AUCs of 0.948 and 0.945, respectively (Supplementary Table 4). Among them, the renin-dependent MLP achieved the highest AUCs, reaching 0.953 in the internal validation and 0.947 in the external validation followed by the

FNN with similarly strong performance. The SVM model yielded the highest MCC (0.745) in the internal validation and achieved the highest ACC (0.906), F1 score (0.878), and MCC (0.814) in the external validation. The RF and GNB models performed particularly well in the internal validation, each showing the highest balanced accuracy (0.882) and F1 score (0.839), respectively, among all models. Although the LR model demonstrated a strong AUC exceeding 0.945, its performance on other evaluation metrics was lower than that of the other models.

For the renin-dependent models (MLP, FNN, and SVM), the probability score intervals between the sensitivity ≥95% and specificity ≥95% thresholds were relatively wide, resulting in broad gray zones (Supplemental Figure 3, Panel b,d,f). Among these, the renin-dependent MLP showed the narrowest gray zone, followed by FNN and then SVM. The renin-dependent SVM model showed the largest intermediate range of indeterminate scores, with a greater proportion of patients falling between rule-out and rule-in thresholds.

Diagnostic performance across varying thresholds

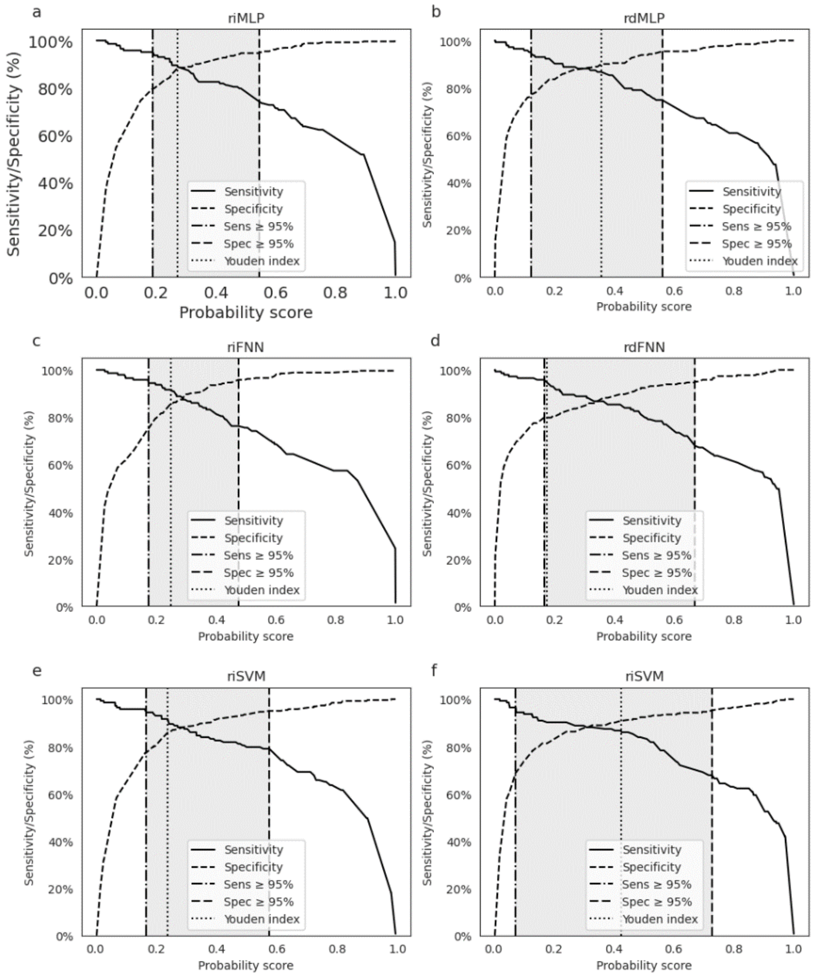

Supplementary Figure 3. Sensitivity and specificity analysis of diagnostic performance across varying thresholds for both renin-independent and renin-dependent SVM, FNN and MLP models. Sensitivity (solid), specificity (dashed) are shown across decision thresholds. Vertical lines denote the Youden-based threshold and those corresponding to 95% sensitivity and 95% specificity. The interval between the 95% sensitivity and 95% specificity thresholds is shown as the gray zone.

Receiver operating characteristic (ROC) curve tables

Across the range of sensitivities from 85% to 95%, both renin-independent and renin-dependent ML models consistently achieved substantially higher specificity than the ARR (Supplementary Tables 5 and 6). For example, at close to 95% sensitivity, the specificity of the ARR was 51.9%, whereas ML models maintained specificities in the range of approximately 74% to 80%. This indicates a more

favorable trade-off between sensitivity and specificity for ML models, which underlies the observed reductions in false-positive results compared with ARR.

Supplementary Table 5: ROC table with 85% to 95% sensitivity for ARR.

Threshold (pmol/mU)	Sensitivity %	Specificity %	Balanced Accuracy %
35.39*	86.0	68.7	77.4
32.45	87.4	66.4	76.9
29.47	88.1	63.7	75.9
26.58	90.2	60.7	75.4
25.57	91.6	60.3	76.0
25.17	92.3	59.2	75.7
23.48	93.0	56.5	74.7
22.16	94.4	55.0	74.7
21.04	95.1	51.9	73.5
20.32	95.8	50.8	73.3

Supplementary Table 6: ROC table with 85% to 95% sensitivity for renin-independent and -dependent models.

Threshold	Sensitivity %	Specificity %	Balanced Accuracy %	Threshold	Sensitivity %	Specificity %	Balanced Accuracy %
riSVM				rdSVM			
0.334	86.0	88.9	87.5	0.448	86.0	91.2	88.6
0.304	87.4	88.5	88.0	0.424*	86.7	90.8	88.8
0.292	88.1	88.2	88.1	0.392	87.4	90.1	88.7
0.272	88.8	87.4	88.1	0.328	88.1	88.9	88.5
0.239*	91.6	85.9	88.7	0.242	90.2	86.3	88.2
0.218	93.0	83.2	88.1	0.162	90.9	81.3	86.1
0.193	93.7	80.2	86.9	0.145	92.3	79.8	86.0
0.166	95.1	77.5	86.3	0.128	93.7	77.9	85.8
0.164	95.8	77.1	86.5	0.101	94.4	73.7	84.0
0.081	96.5	60.7	78.6	0.074	96.5	69.1	82.8
riFNN				rdFNN			
0.340	86.0	90.5	88.2	0.419	85.3	89.7	87.5
0.295	87.4	89.7	88.6	0.363	86.7	88.2	87.4
0.280	88.8	87.4	88.1	0.311	88.1	85.9	87.0
0.267	89.5	86.6	88.1	0.307	88.8	85.1	87.0
0.259	90.9	86.3	88.9	0.264	89.5	83.6	87.4
0.249*	91.6	85.9	88.7	0.221	90.9	82.1	86.5
0.228	92.3	82.8	87.6	0.217	91.6	81.3	86.5
0.216	93.7	81.7	87.7	0.194	93.0	80.5	86.8
0.174	94.4	79.4	84.8	0.164*	95.8	79.8	87.8
0.172	95.8	75.2	85.5	0.129	96.5	77.5	87.0
riMLP				rdMLP			
0.323	86.0	89.7	87.9	0.393	85.3	90.5	87.9
0.306	87.4	88.9	88.2	0.356*	86.7	90.1	88.4
0.273	88.8	88.5	88.7	0.315	88.1	88.5	88.3
0.270*	89.5	88.2	88.8	0.226	90.2	85.8	88.0
0.251	90.9	85.9	88.4	0.182	92.3	83.6	87.9
0.245	92.3	84.4	88.3	0.168	93.0	82.1	87.5
0.227	93.0	83.2	88.1	0.132	93.7	77.9	85.8
0.205	93.7	80.9	87.3	0.122	94.4	77.5	85.9
0.188	95.1	79.8	87.4	0.121	95.1	77.1	86.1
0.148	95.8	74.8	85.3	0.110	95.8	76.0	85.9

FNN: feedforward neural networks; GNB: Gaussian naïve Bayes; MLP: multi-layer perceptron; LR: logistic regression; SVM: support vector machine; RF: random forest.

Post-test probability across pre-test prevalence and decision thresholds

Across both renin-independent and renin-dependent models, post-test probability increased with increased pre-test prevalence from low- to intermediate- and high-prevalence settings (Supplementary Figure 4). This trend was observed consistently across all six models, while different cut-offs (Youden, 50% sensitivity, and 95% sensitivity) produced distinct probability curves within each model, indicating that both baseline prevalence and threshold selection influenced post-test interpretation.

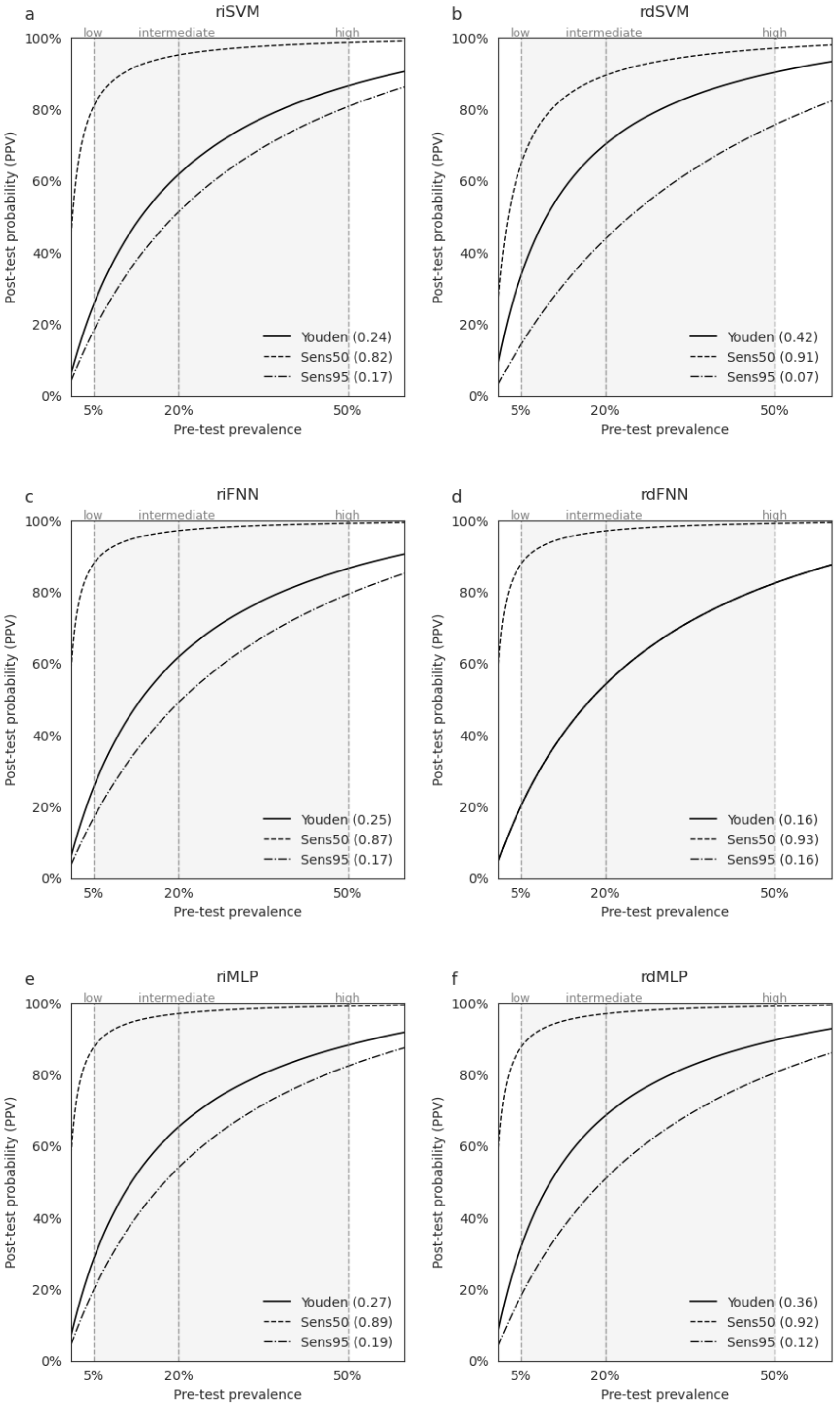

Supplementary Figure 4. Positive predictive probability is shown as a function of pre-test prevalence for both renin-independent and renin-dependent SVM, FNN and MLP models across different decision thresholds, including those defined

by the Youden index, 95% sensitivity, and 50% sensitivity. Performance is illustrated at clinically relevant pre-test probabilities of 5%, 20%, and 50%, indicated by vertical dashed lines, corresponding to low-, intermediate-, and high-probability clinical settings, respectively.

As shown in Supplemental Table 7, across all prevalence scenarios, ML models consistently reduced the number of false-positive results compared with the ARR at matched sensitivities. At a prevalence of 5%, the ARR yielded 457 false positives per 1,000 screened patients, whereas ML models reduced this to between 163 and 265 per 1,000 patients. At prevalences of 20% and 50%, similar patterns were observed, with reductions ranging from approximately 86 to 223 fewer false positives per 1,000 patients.

These reductions were broadly consistent across both renin-independent and renin-dependent models, indicating that the observed improvements were not confined to a specific model type but reflect a general advantage of ML-based approaches in improving specificity at high sensitivity thresholds.

Supplementary Table 7: Absolute numbers of true positives (TP), false positives (FP), false negatives (FN), and true negatives (TN) per 1,000 screened hypertensive patients for the ARR and all renin-independent and –dependent models at different prevalence levels (5%, 20%, and 50%). All values are calculated using thresholds corresponding to 95% sensitivity.

	Prevalence	Specificity	PPV	NPV	TP per 1000	FP per 1000	FN per 1000	TN per 1000
ARR	5%	51.9%	0.094	0.995	48	457	2	493
	20%	51.9%	0.331	0.977	190	385	10	415
	50%	51.9%	0.664	0.914	476	241	25	260
riSVM	5%	77.5%	0.182	0.997	48	214	2	736
	20%	77.5%	0.514	0.984	190	180	10	620
	50%	77.5%	0.809	0.941	476	113	24	387
riFNN	5%	75.2 %	0.169	0.997	48	236	2	714
	20%	75.2 %	0.491	0.986	192	198	8	602
	50%	75.2 %	0.794	0.947	479	124	21	376
riMLP	5%	79.8%	0.198	0.997	48	192	2	758
	20%	79.8%	0.540	0.985	190	162	10	639
	50%	79.8%	0.825	0.942	476	101	25	399
rdSVM	5%	69.1 %	0.141	0.997	48	294	2	656
	20%	69.1 %	0.438	0.988	193	247	7	553
	50%	69.1 %	0.757	0.952	483	155	17	345
rdFNN	5%	79.8%	0.200	0.997	48	192	2	758
	20%	79.8%	0.542	0.987	192	162	8	638
	50%	79.8%	0.826	0.950	479	101	21	399
rdMLP	5%	77.1%	0.179	0.997	48	218	2	732
	20%	77.1%	0.509	0.984	190	183	10	617
	50%	77.1%	0.806	0.940	476	115	24	385

Model calibration

The collaboration plots for both renin-independent and renin-dependent models are presented in Supplementary Figure 5, Panel a and b. Most renin-independent models demonstrated close agreement between predicted and observed probabilities, with FNN, MLP, LR, and SVM showing the best calibration with the Brier scores from 0.088 to 0.089. GNB displayed the greatest deviation with the Brier score of 0.105, while RF slightly underestimated probabilities in the mid-range with the Brier score of 0.096.

The collaboration curves for renin-dependent models - FNN, GNB, MLP, LR, RF, SVM - demonstrated slightly improved calibration compared with the renin-independent models, with MLP and FNN yielding the lowest Brier scores (0.086-0.087). Minor deviations from perfect calibration were observed for GNB with a Brier score of 0.099 and LR with a Brier score of 0.093.

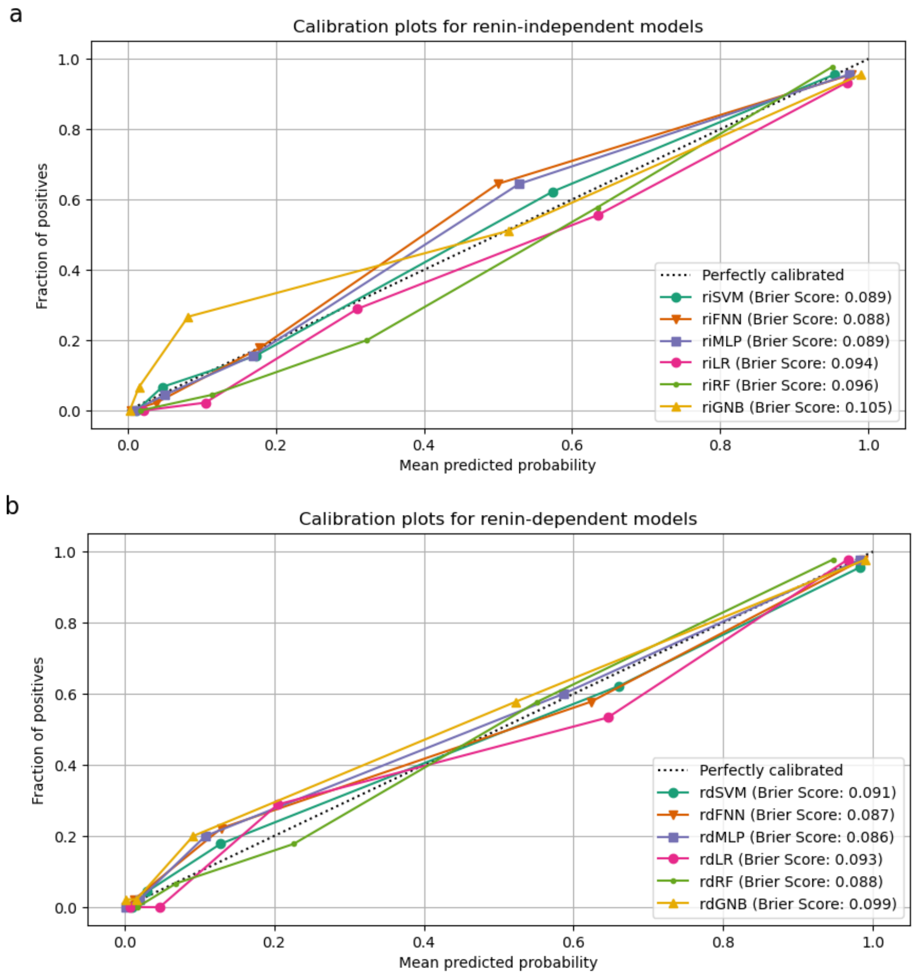

Supplementary Figure 5. Collaboration plots of FNN, GNB, MLP, LR, RF, and SVM models without renin and with renin. Models were trained on 80% combined datasets, SPISCA and PROSALDO. And validated on 20% combined datasets for testing set. The dotted line represents perfect calibration. Panel a: Collaboration plots for renin-independent models. Panel b: Collaboration plots for renin-dependent models.

Model concordance

Supplementary Figure 6 showed the agreement of predicted probability scores among the renin-independent and renin-dependent SVM, FNN and MLP models. The closest agreement was observed between the FNN and MLP models, with Spearman correlation coefficients of 0.992 for both the renin-independent (panel c) and renin-dependent (panel f) models. Moderate but still high concordance was seen between the SVM and MLP, with correlation coefficients of 0.958 for the renin-independent model (panel b) and 0.935 for the renin-dependent model (panel e). The lowest, yet still strong, correlations were found between the SVM and the FNN, with coefficients of 0.949 in the renin-independent model (panel a) and 0.928 in the renin-dependent model (panel d). Overall, these plots indicate that all three model types generate very similar probability rankings in both the renin-independent and renin-dependent feature sets.

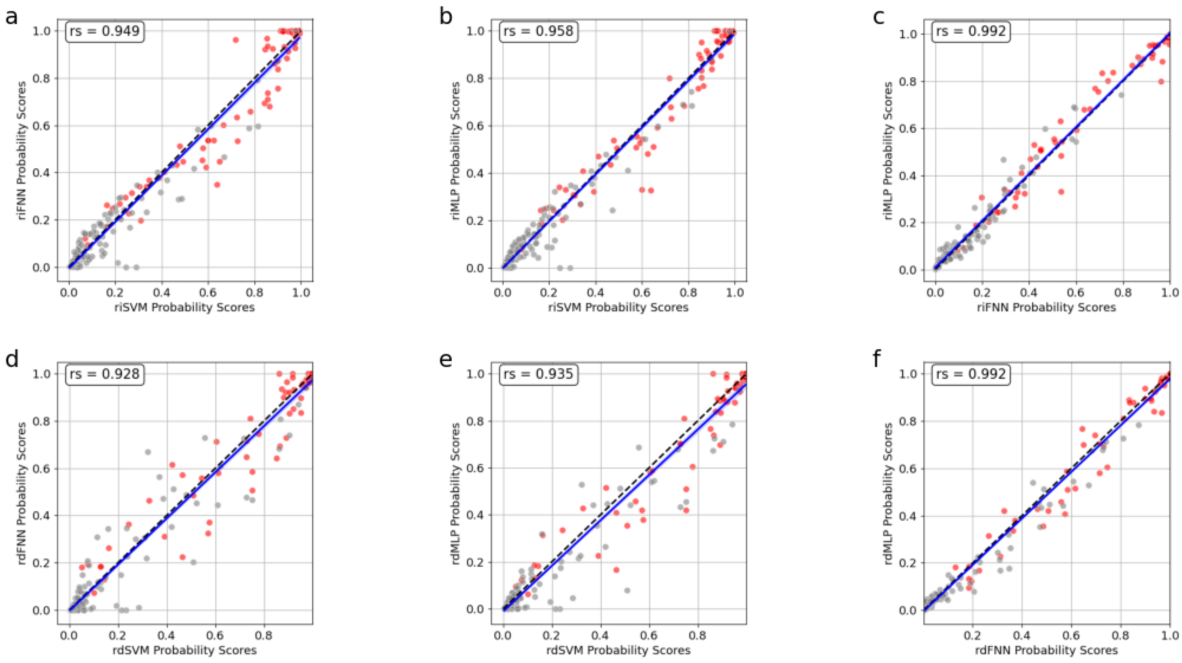

Supplementary Figure 6. Relationships of probability scores for SVM, FNN, and MLP models with renin and without renin (○ patients without PA; ○ patients with PA). Models were trained on 80% combined datasets, SPISCA and PROSALDO. And validated on 20% combined datasets for testing set. Dotted lines display linear fits of relationships and associated Spearman's rank correlation coefficients (rs). The blue line represents the best fit.

TRIPOD-AI Checklist

**Supplementary Table 8:** TRIPOD+AI checklist for the reporting of prediction model studies.				
Section/topic	Item	Development/ Evaluation*	Checklist item	Reported on page
Title				
Title	1	D;E	Identify the study as developing or evaluating the performance of a multivariable prediction model, the target population, and the outcome to be predicted	1
Abstract				
Abstract	2	D;E	See TRIPOD-AI for Abstracts checklist	3
Introduction				
Background	3a	D;E	Explain the healthcare context (including whether diagnostic or prognostic) and rationale for developing or evaluating the prediction model, including references to existing models	4,5
	3b	D;E	Describe the target population and the intended purpose of the prediction model in the context of the care pathway, including its intended users	5
	3c	D;E	Describe any known health inequalities between sociodemographic groups	NA
Objectives	4	D;E	Specify the study objectives, including whether the study describes the development or validation of a prediction model (or both)	5,6
Methods				
Data	5a	D;E	Describe the sources of data separately for the development and evaluation datasets (eg, randomised trial, cohort, routine care or registry data), the rationale for using these data, and representativeness of the data	5,6,8
	5b	D;E	Specify the dates of the collected participant data, including start and end of participant accrual; and, if applicable, end of follow-up	16
Participants	6a	D;E	Specify key elements of the study setting (eg, primary care, secondary care, general population) including the number and location of centers	15-16
	6b	D;E	Describe the eligibility criteria for study participants	15-16
	6c	D;E	Give details of any treatments received, and how they were handled during model development or evaluation, if relevant	16-19
Data preparation	7	D;E	Describe any data pre-processing and quality checking, including whether this was similar across relevant sociodemographic groups	20
Outcome	8a	D;E	Clearly define the outcome that is being predicted and the time horizon, including how and when assessed, the rationale for choosing this outcome, and whether the method of outcome assessment is consistent across sociodemographic groups	16-19
	8b	D;E	If outcome assessment requires subjective interpretation, describe the qualifications and demographic characteristics of the outcome assessors	NA
	8c	D;E	Report any actions to blind assessment of the outcome to be predicted	NA
Predictors	9a	D	Describe the choice of initial predictors (eg, literature, previous models, all available predictors) and any pre-selection of predictors before model building	5,6
	9b	D;E	Clearly define all predictors, including how and when they were measured (and any actions to blind assessment of predictors for the outcome and other predictors)	16,17,19
	9c	D;E	If predictor measurement requires subjective interpretation, describe the qualifications and demographic characteristics of the predictor assessors	NA
Sample size	10	D;E	Explain how the study size was arrived at (separately for development and evaluation), and justify that the study size was	21

			sufficient to answer the research question. Include details of any sample size calculation	
Missing data	11	D;E	Describe how missing data were handled. Provide reasons for omitting any data	No missing data
Analytical methods	12a	D;E	Describe how the data were used (eg, for development and evaluation of model performance) in the analysis, including whether the data were partitioned, considering any sample size requirements	5,6,8,9 Figure 1
	12b	D	Depending on the type of model, describe how predictors were handled in the analyses (functional form, rescaling, transformation, or any standardisation)	19-21
	12c	D	Specify the type of model, rationale†, all model building steps, including any hyperparameter tuning, and method for internal validation	19-21
	12d	D;E	Describe if and how any heterogeneity in estimates of model parameter values and model performance was handled and quantified across clusters (eg, hospitals, countries). See TRIPOD-Cluster for additional considerations‡	Planned in a next publication on robustness
	12e	D;E	Specify all measures and plots used (and their rationale) to evaluate model performance (eg, discrimination, calibration, clinical utility) and, if relevant, to compare multiple models	20,21 Sup 6-13
	12f	E	Describe any model updating (eg, recalibration) arising from the model evaluation, either overall or for particular sociodemographic groups or settings	NA
	12g	E	For model evaluation, describe how the model predictions were calculated (eg, formula, code, object, application programming interface)	19-22
Class imbalance	13	D;E	If class imbalance methods were used, state why and how this was done, and any subsequent methods to recalibrate the model or the model predictions	20
Fairness	14	D;E	Describe any approaches that were used to address model fairness and their rationale	NA
Model output	15	D	Specify the output of the prediction model (eg, probabilities, classification). Provide details and rationale for any classification and how the thresholds were identified	7-11
Training vs evaluation	16	D;E	Identify any differences between the development and evaluation data in healthcare setting, eligibility criteria, outcome, and predictors	6
Ethical approval	17	D;E	Name the institutional research board or ethics committee that approved the study and describe the participant informed consent or the ethics committee waiver of informed consent	16
Open science				
Funding	18a	D;E	Give the source of funding and the role of the funders for the present study	2
Conflicts of interest	18b	D;E	Declare any conflicts of interest and financial disclosures for all authors	23
Protocol	18c	D;E	Indicate where the study protocol can be accessed or state that a protocol was not prepared	21-22
Registration	18d	D;E	Provide registration information for the study, including register name and registration number, or state that the study was not registered	16 & 21-22
Data sharing	18e	D;E	Provide details of the availability of the study data	22
Code sharing	18f	D;E	Provide details of the availability of the analytical code§	22
Patient and public involvement				
Patient and public involvement	19	D;E	Provide details of any patient and public involvement during the design, conduct, reporting, interpretation, or dissemination of the study or state no involvement	NA
Results				

Participants	20a	D;E	Describe the flow of participants through the study, including the number of participants with and without the outcome and, if applicable, a summary of the follow-up time. A diagram may be helpful	5-6, 15-19 Figure 1
	20b	D;E	Report the characteristics overall and, where applicable, for each data source or setting, including the key dates, key predictors (including demographics), treatments received, sample size, number of outcome events, follow-up time, and amount of missing data. A table may be helpful. Report any differences across key demographic groups	5-6,15-19 Sup 2
	20c	E	For model evaluation, show a comparison with the development data of the distribution of important predictors (demographics, predictors, and outcome)	5-6, Sup 2
Model development	21	D	Specify the number of participants and outcome events in each analysis (eg, for model development, hyperparameter tuning, model evaluation)	5-8,16-21 Figure 1
Model specification	22	D	Provide details of the full prediction model (eg, formula, code, object, application programming interface) to allow predictions in new individuals and to enable third party evaluation and implementation, including any restrictions to access or reuse (eg, freely available, proprietary)¶	19-22
Model performance	23a	D;E	Report model performance estimates with confidence intervals, including for any key subgroups (eg, sociodemographic). Consider plots to aid presentation	6-11, Sup 2-13
	23b	E	If examined, report results of any heterogeneity in model performance across clusters. See TRIPOD-Cluster for additional details‡	NA
Model updating	24	E	Report the results from any model updating, including the updated model and subsequent performance	NA
Discussion				
Interpretation	25	D;E	Give an overall interpretation of the main results, including issues of fairness in the context of the objectives and previous studies	11
Limitations	26	D;E	Discuss any limitations of the study (such as a non-representative sample, sample size, overfitting, missing data) and their effects on any biases, statistical uncertainty, and generalisability	13-14
Usability of the model in the context of current care	27a	D;	Describe how poor quality or unavailable input data (eg, predictor values) should be assessed and handled when implementing the prediction model	Since only 5-7 features are required there should be no missing data
	27b	D;	Specify whether users will be required to interact in the handling of the input data or use of the model, and what level of expertise is required of users	NA
	27c	D;E	Discuss any next steps for future research, with a specific view to applicability and generalisability of the model	12-13

TRIPOD=Transparent Reporting of a multivariable prediction model for Individual Prognosis Or Diagnosis; AI=artificial intelligence.
**D=items relevant only to the development of a prediction model; E=items relating solely to the evaluation of a prediction model; D;E=items applicable to both the development and evaluation of a prediction model.*
†Separately for all model building approaches.
‡TRIPOD-Cluster is a checklist of reporting recommendations for studies developing or validating models that explicitly account for clustering or explore heterogeneity in model performance (eg, at different hospitals or centers)
§Relates to the analysis code, for example, any data cleaning, feature engineering, model building, and evaluation.
¶Relates to the code to implement the model to get estimates of risk for a new individual
